# CFD simulation of updrafts initiated by a vertically directed jet fed by the heat of water vapor condensation

**DOI:** 10.1038/s41598-022-13185-2

**Published:** 2022-06-07

**Authors:** Magomet T. Abshaev, Ali M. Abshaev, Andrey A. Aksenov, Iuliia V. Fisher, Alexander E. Shchelyaev, Abdulla Al Mandous, Youssef Wehbe, Reyad El-Khazali

**Affiliations:** 1 Hail Suppression Research Center “Antigrad”, 198 Chernishevsky Street, Nalchik, 360004 Russia; 2 Engineering Company TESIS, 18 Yunnatov str., 7th floor, office 705, Moscow, 127083 Russia; 3 National Center of Meteorology, P.O. Box: 4815, Abu Dhabi, UAE; 4 Department of Electrical Engineering and Computer Science, Khalifa University of Science and Technology, P.O. Box: 127788, Abu Dhabi, UAE; 5 Joint Institute for High Temperatures, Russian Academy of Sciences, 13 Izhorskaya st, Moscow, 125412 Russia

**Keywords:** Environmental sciences, Planetary science, Mathematics and computing, Applied physics, Condensed-matter physics, Fluid dynamics

## Abstract

This paper presents the results of the development of a mathematical model and numerical simulation of the ascent in the atmosphere of a vertically directed jet fed by the heat of condensation of water vapor on a hygroscopic aerosol introduced into the jet at the start. The possibility of creating artificial convective clouds depending on jet parameters, condensation heat value and vertical profiles of wind speed, air temperature and humidity has been evaluated. Numerical experiments showed that the motion of a high-speed and high-temperature jet in the atmosphere has a complex turbulent nature. As the jet ascends, it expands, losing superheat and velocity. The temperature of the jet decreases faster than the velocity, so the jet rises slightly above the level at which its superheat disappears. The jet's ascent height increases as the humidity of the air and the vertical temperature gradient increase. Wind causes the jet to deform, bend, and decrease the height of ascent. Feed the jet with condensation heat results in a significant increase in jet lift height. This is particularly effective in the case of introducing into the jet two-layer NaCl/TiO_2_ nanoaerosol, which is capable of absorbing water vapor in an amount significantly greater than its mass. The simulation results are encouraging in the possibility of creating artificial updrafts that can lead to the formation of convective clouds and precipitation on days with favorable atmospheric conditions, when wind speed in the sub-cloud layer is < 6 m/s, air humidity is > 65%, and the temperature lapse rate is > 7.5 °C/km.

## Introduction

According to the United Nations, about 60% of the world's population lacks the fresh water they need to live and sustain their economy (Manzoor 2020). Global warming and increasing water consumption are exacerbating this problem, especially in arid and semi-arid regions where traditional water sources (rivers, lakes, rainfall, and readily available groundwater) can no longer meet the demand for fresh water^1–4^. This is forcing the development of technologies for seawater desalination, deep groundwater extraction on land and sea, iceberg towing, treatment and reuse of municipal wastewater, irrigation system drainage water, and ballast water from tanker and ship holds.

One of the relatively cheap and environmentally friendly methods of replenishing fresh water resources is harvesting of atmospheric moisture from fogs and artificial rain enhancement. The atmosphere contains about $1.3\times {10}^{13}$ tons of water vapor. This content is systematically updated due to the global hydrological cycle of condensation-precipitation-evaporation with a periodicity of 40–45 days^5^.

The collection of water from fog involves the deposition of droplets on polypropylene meshes^6^ or three-dimensional spacer fabrics^7^. These systems, depending on the fog duration and water content, can produce from 2 to 20 L of water per day for each sq. m. of mesh^8^.

An artificial precipitation enhancement is carried out through clouds seeding using condensation or glaciogenic nuclei released from aircraft, rocket or ground generators. These methods can increase precipitation by up to 20% of the annual rate, depending on cloud resources, their type and characteristics of seeding facilities^9,10^.

However, in arid regions, the number of cloudy days and cloud resources are very limited^11,12^. Therefore, within the framework of grant APP-REP-2017–02,120 UAE Research Program for Rain Enhancement Science, studies are being carried out on the possibility of initiating updrafts and creating artificial clouds and precipitation^13–15^.

It has long been noticed that over powerful natural heat sources (forest and other large fires, active volcanoes, mountain tops warmed by the sun), cumulus clouds, called Pyro-clouds, are formed. The formation of convective clouds, called Industry-clouds, was also noted over the "heat islands" created by megacities, nuclear power plants, large oil refineries and thermal power plants. The reason for the development of Pyro-clouds and Industry-clouds is the heating of local areas of the surface atmosphere and the formation of thermals. Under some favorable atmospheric conditions, these thermals can combine into updraft jets, rising to the level of condensation, and lead to the formation of cumulus and even cumulonimbus clouds.

Almost all earlier attempts to create artificial clouds and precipitation envisaged heating the surface atmosphere using various heat sources. Even in antiquity, shamans in South America and Equatorial Africa created artificial fires in the prairies and savannas to cause rain in drought (Dessens 1969). In 1960–1970, in France and the USSR, a number of studies were carried out on the possibility of creating artificial clouds and precipitation using the so-called meteotrons, in which oil products were abundantly burned such as:

1. Meteotrons used in France^16,17^, containing 100 or more jet burners located on square area with side of 125 m^16^ or a hexagon with a radius of 36 m or a three-link spiral^18^. Depending on the number of operating burners, these meteotrons burned from 60 to 105 tons/hour of gas oil and their power reached from 700 to 1000 MW.
2. Meteotron of the Institute of Geology and Geophysics of the Siberian Branch of the Russian Academy of Sciences, which contained 60 jet flamethrowers located along the perimeter of an octagon with a side of 53 m. When burning about 430 tons/hour of diesel fuel, its power reached 5000–6000 MW. The updrafts and black smoke in some of the 8 experiments rose to heights of up to 3 km.
3. Meteotrons of the Chelyabinsk Polytechnic Institute (8 types), containing from 10 to 100 centrifugal nozzles with a diesel fuel consumption of 7 to 30 tons/hour, developed a capacity of 80 to 400 MW^19,20^.
4. Meteotrons of the Institute of Applied Geophysics of the USSR Goskomhydromet with 4 and 10 jet engines, as well as the Supermeteotron with 6 aircraft engines, had a power of 300–500 MW^21^.

The experiments that were carried out in France on the Lannemesan plateau that lasted from 5 to 40 min^16,18^ showed that the updrafts created by individual burners at a height of 10 m merged into one common jet, which had a radius of about 70 m, an average temperature rise over ambient air Δ*T* = 50 °C and a lifting speed *W* = 3–4 m/s. It was also observed that as the jet radius *R* increases, Δ*T* decreases, while *W* reaches its maximum at a height of *H* ≈ 150–300 m, and above that it decreases to zero. At vertical temperature lapse rate of *γ* = 8–10 °C/km and wind speeds near the ground and at heights up to 5 m/s, artificial ascending currents reached an altitude of 800–1300 m and pierced temperature inversion layers up to 150 m thick, located below 1000 m^18^. In none of the 11 experiments the torch did rise above 1600 m and did reach the level of condensation.

It is worth mentioning that^21^ performed 36 experiments and obtained the following results:

- 4 cases—no effect (Anticyclonic conditions with stable atmospheric stratification, wind speed near the ground 9–12 m/s);
- 18 cases—formation of small cumulus clouds above the meteotron and its leeward side or the development of existing ones (Anticyclonic conditions, retarding layers near or above the condensation level with a wind speed 3–6 m/s);
- 4 cases—development of natural Cu Hum to the Cu Cong stage (periphery of anticyclones or cyclones, the presence of positive CAPE, shallow retarding layers near the condensation level);
- 10 cases—development of natural Cu Hum or Cu Cong to the Cb stage with precipitation (intramass development in the rear of cyclones (5 cases) and the influence of blurred cold fronts, when in a large layer above the condensation level, there is a positive CAPE with isothermal layers of small thickness. In two cases, there were no retarding layers. The wind at the ground did not exceed 7–8 m/s).

At low wind speeds, the buoyancy jet rises to an altitude of 600–800 m and only under certain favorable conditions it reaches 1000–1200 m^21^. This is confirmed by the results of the theoretical modeling^22^.

In most cases, the jet overheating Δ*T* and buoyancy rapidly decrease with height and the initial energy of the jet turns out to be insufficient to overcome the layers of temperature and humidity inversion.

Based on theoretical and experimental studies of the performance of various physical principles for stimulating convection in a cloudless atmosphere, a new method of creating updrafts, artificial clouds, and precipitation has been proposed^14^ using a vertically directed buoyancy jet saturated with three types of hygroscopic aerosol with different hygroscopic points,i.e., for *h*_1_ < 40%, 41 < *h*_2_ < 70%, and 71 < *h*_3_ |< 80%. In this case, the jet serves to initiate ascending flows, and condensation of water vapor on a coarsely dispersed hygroscopic aerosol can lead to energy replenishment and an increase in the buoyancy of ascending flows due to the release of condensation heat.

In this case, convection is stimulated by the following factors:

1. Kinetic energy of the buoyancy jet creates an initial convection impulse;
2. High initial jet temperature ensures the buoyancy of the upward flow;
3. Release of water vapor condensation heat on aerosol introduced into the jet, as well as the energy of solar radiation absorbed by the aerosol, enhances the ascending flow;
4. A convective cloud is formed, after reaching condensation level, which develops due to the release of condensation heat, and can turn into a cumulonimbus cloud giving rainfall.

The main objectives of this work are: (a) to develop a mathematical model and numerical simulation of the motion in the atmosphere of a vertically directed jet fed by the heat of condensation of water vapor on a hygroscopic aerosol, and (b) to evaluate the possibility of creating artificial convective clouds depending on the parameters of the jet, the value of feeding and vertical profiles of wind speed, air temperature and humidity.

## Methodology and model description

### Evaluation of energy supply jet with condensation heat

The amount of additional energy that the jet will receive due to the release of condensation heat depends on the hygroscopic properties of the aerosol, which determine at what air humidity and how much water vapor can condense on the aerosol particle. When air humidity exceeds the hygroscopic point of the aerosol substance, a saturated solution droplet is first formed on the aerosol particle, over which the water vapor pressure ES is lower than the ambient pressure E. This leads to further growth of the droplet due to diffusion of water vapor until Δ*E*_*S*_ = (*E*_*∝*_—*E*_*S*_) > 0.

It is known that the pressure of water vapor over the drop depends on the air temperature *T*, the radius of curvature of the drop *r*, the electric charge *e* and the concentration of aqueous solution *C* in the drop formed on the hygroscopic particle. Approximately, this dependence can be represented as:

1 $$E={E}_{\infty }\left(1+\frac{{c}_{r}}{r}-\frac{{c}_{q}}{{r}^{4}}-\frac{{n}_{a}}{{n}_{a}+{n}_{w}}\right).$$

where *c*_*r*_, *c*_*q*_ are some constants characterizing the properties of water vapor, surface tension at the water–vapor boundary, the number and magnitude of elementary charges in a drop; *n*_*a*_ = *m*_*a*_*/μ*_*a*_ and *n*_*w*_ = *m*_*w*_*/μ*_*w*_ are the numbers of moles of the hygroscopic substance and water dissolved in the drop, respectively; *m*_*a*_ and *m*_*w*_ are their masses; *μ*_*a*_ and *μ*_*w*_ are their molar mass, respectively.

At an initial droplet size of more than 1 μm, the effect of size and electric charge becomes negligible, and at a given temperature, the value of *E* depends only on the concentration of the solution:

2 $$E={E}_{\infty }\left(1-\frac{{n}_{a}}{{n}_{a}+{n}_{w}}\right)={E}_{\infty }\frac{{n}_{w}}{{n}_{a}+{n}_{w}}.$$

For saturated solutions we have:

3 $$\left\{\begin{array}{c}\frac{{E}_{s}}{{E}_{\infty }}={\left(\frac{{n}_{w}}{{{n}_{a}+n}_{w}}\right)}_{S}={C}_{C} \\ \Delta {E}_{S}={E}_{\infty }-{E}_{s}={E}_{\infty }\frac{{n}_{a}}{{{n}_{a}+n}_{w}}\\ \frac{{\Delta E}_{s}}{{E}_{\infty }}=\frac{{n}_{a}}{{n}_{a}+{n}_{w}}\end{array}\right.$$

As the droplet grows further, the concentration of the solution *n*_*a*_*/n*_*w*_ decreases by diluting the solution in proportion to the increase in droplet mass:

4 $$\frac{{\left(\frac{{n}_{a}}{{{n}_{a}+n}_{w}}\right)}_{t}}{{\left(\frac{{n}_{w}}{{{n}_{a}+n}_{w}}\right)}_{S}}=\frac{{m}_{s}}{{m}_{t}}.$$

Drop growth stops at *n*_*w*_ >  > *n*_*a*_ when water vapor pressure drops to equilibrium pressure. In this case, according to Raoul's empirical law for weak solutions, we have:

5 $${\left(\frac{{n}_{a}}{{n}_{w}}\right)}_{t}=\frac{{\Delta E}_{s}}{{E}_{\infty }}={C}_{s}.$$

Taking into account (3) and (5) we obtain from expression (4):

6 $${m}_{t}={m}_{s}\frac{{C}_{c}}{{C}_{s}}.$$

where *m*_*s*_ is the drop mass of a saturated solution; *m*_*t*_ is the final mass of the drop, to which it can grow by the time *t*, when the water vapor pressure over the drop approaches the equilibrium one; *C*_*C* _= *E*_*S*_/*E*_*∝*_ is the ratio of water vapor pressure over a drop of saturated solution to pressure over a flat surface of distilled water; *C*_*S*_ = (∆*Es*)/*E*_*∞*_ is the ratio of the difference between the indicated pressures to the pressure above the flat surface of distilled water.

The mass of a drop of saturated solution can be represented through the mass of a dry aerosol particle and the amount of condensed water in the form:

7 $$m_{s} \, = \,m_{a} \, + \,m_{w} \, = \,m_{a} ({\text{1}}\, + \,\kappa )$$

where *κ* is the ratio of the mass of the solvent to the mass of the dissolved substance.

Substituting this value *m*_*s*_ into (6) we obtain the final mass of the drop depending on the mass of the aerosol particle:

8 $${m}_{t}={m}_{a}\left(1+k\right)\frac{{C}_{c}}{{C}_{s}}$$

The amount of condensed water is

9 $${m}_{w}={m}_{t}-{m}_{a}$$

Substituting the value of *m*_*t*_ from (8) to (9) we get:

10 $${m}_{w}={m}_{a}\left[\left(1+\kappa \right)\frac{{C}_{c}}{{C}_{s}}-1\right]$$

The ratio of water and solute mass at the equilibrium pressure of water vapor *k*_1_ is equal to:

11 $${k}_{1}=\frac{{m}_{w}}{{m}_{a}}=\left[\left(1+\kappa \right)\frac{{C}_{c}}{{C}_{s}}-1\right]$$

The value *κ* can be found in reference books on saturated solutions. For example, at 20 °C in a saturated solution of NaCl, there are 37.1 g of NaCl per 100 g of water. The value *κ* = 100/37.1 = 2.801. Experimental values of *C*_*C*_ and *C*_*s*_ for different substances are given in reference books and textbooks (e.g.^23^. For example, for NaCl *C*_*C*_ = 0.78, *C*_*S*_ = 0.22; for CaCl_2_ *C*_*C*_ = 0.65; *C*_*S*_ = 0.35.

According to (11) an aerosol particle of NaCl can condense *k*_1_ = 12.48 times its dry mass. By analogy, for CaCl_2_ we have *κ* = 1000/745 = 1.34 and *k*_1_ = 3.34, and for (NH_2_)_2_CO *κ* = 1000/518 = 1.93, *k*_1_ = 6.17.

Each NaCl aerosol particle with diameter of 10 µm and mass *m*_*a*_ = 1.133·10^–9^ g can condense *m*_*w*_ = 3.174·10^–9^ g of water and form a drop of saturated solution with mass *m*_*s*_ = 4.31·10^–9^ g. In this case, its diameter will be equal to *d*_*S*_ = 19 μm, which coincides with the data that the ratio of the diameter of a drop of saturated solution to the diameter of dry NaCl particles is *d*_*S*_/*d*_*a*_ = 1.9^24^.

When the air humidity is above the hygroscopic point (75.3%), drops of a saturated NaCl solution will grow until the equilibrium pressure of water vapor is established and the final mass of the drops reaches the value *m*_t_ = 13.48*m*_a_ ≈ 15.27·10^–9^ g. This means that the droplet diameter will reach *d*_*t*_ = 30.25 µm. This is consistent with the data of laboratory experiments^25^, according to which NaCl particles weighing 10^–9^ g (*d*_*a*_ = 9.6 μm) form drops with a diameter of 30 μm for several seconds.

The introduction of *N* = 2·10^11^ s^-1^ of NaCl particles with diameter of 10 μm and total mass of about 230 g/s into the jet can lead to condensation of the total amount of water *M*_*w*_, equal to

$${M}_{w}=N{m}_{a}k1=2\cdot {10}^{11}\cdot 1.133\cdot {10}^{-9}\cdot 12.47 \approx 2825\mathrm{ g}/\mathrm{s}.$$

This will lead to the release of an amount of heat equivalent to the power source:

$${Q}_{2}={M}_{wt}\cdot q$$ = 2.825⋅2260 ≈ 6.39 MJ/s = 6.39 MW.

The introduction of NaCl/TiO_2_ micro powder with *k*_1_ = 295 into the jet at 230 g/s^26^ can lead to the condensation of water vapor in the amount

$${M}_{wt}^{\prime}=N{\cdot {m}_{a}\cdot k}_{1}=2\cdot {10}^{11}\cdot 1.133\cdot {10}^{-9}\cdot 295$$ $$\approx 66.8\mathrm{ kg}/\mathrm{s}.$$

In this case, the jet feed power will be:

$${P}_{c}={M}_{wt}^{\prime}\cdot q$$ = 66.8⋅2260 ≈ 151 MJ/s = 151 MWt.

This means that the use of NaCl/TiO_2_ aerosol instead of pure NaCl will increase the amount of feeding the jet with the heat of condensation *P*_*C*_ by about 24 times and significantly increase the potential for creating artificial clouds.

### Jet motion simulation in atmosphere

The simulation of the motion of a vertically directed high-temperature jet saturated with a hygroscopic aerosol in the atmosphere, having an initial temperature *T*_*0*_, second mass flow rate *M*_*0*_, and an initial velocity *W*_*0*_ was carried out in a 3D computational fluid dynamics package suite FlowVision^27,28^. A rising upward jet is simulated by gradually expanding and increasing its mass due to the ambient air entrainment. The vertical speed and temperature of the jet decrease as it rises, but the heat of water vapor condensation, as well as the heat of solar radiation absorbed by the aerosol, maintain jet overheating Δ*T* and buoyancy. Observe that the jet radius, temperature, vertical speed, and lift height depend on:

- Jet parameters at the jet engine outlet (Δ*T*_0_, *W*_0_, *M*_0_);
- the amount of energy replenishment by the heat of water vapor condensation *P*_*C*_ and the heat of solar radiation absorbed by the aerosol *P*_*S*_ (in this work, the influence of, *P*_*S*_, is not taken into account);
- Characteristics of the atmosphere: air temperature and humidity, wind speed and their vertical gradients, as well as the thickness and depth of the retarding layers (in this work, the effect of the retarding layers is not considered).

Under favorable atmospheric conditions, such as weak wind, high air humidity, presence of convective instability, and shallow inversion layers, the upward flow initiated by the jet stream can reach the level of natural condensation and lead to the formation of an artificial convective cloud and the formation of precipitation.

### Working substances

The task uses three substances with the following characteristics:

- atmospheric air with molar mass *m*_*a*_ = 0.028964 kg/mol and density *ρ*_*a*_;
- water vapor having a molar mass *m*_*w*_ = 0.018 kg/mol and density *ρ*_*w*_;
- Reactive gases are a mixture of air and combustion products of jet fuel, having a molar mass *m*_*c*_ = 0.0285 kg/mol and density *ρ*_*c*_.

It is assumed that the densities of these gases change according to the ideal gas law, due to small differences (no more than 1%), and are considered the same at equal temperatures and pressures. Only the differences in viscosity and specific heat capacity of air and combustion products are taken into account. Differences in molar mass are taken into account for determining the jet lift (buoyancy). The hygroscopic aerosol is modeled as an impurity entrained by gases.

### Geometric model

The mathematical problem is divided into two parts: modeling a high-speed jet near the engine and a convective jet in a free atmosphere. The engine jet is modeled in a compressible formulation in the computational domain, which is parallelepiped with dimensions of 70 × 70 × 35 m. The engine nozzle is a cylinder with a diameter of 1.2 m and a height of 0.7 m. A convective jet in the atmosphere at a certain distance from the nozzle is modeled in the formulation of large-scale atmospheric currents in windless and crosswinds versions, i.e.:

- in a windless situation for setting up the model and some comparative calculations of two-dimensional axis symmetric version of a 4-degree sector of a cylinder with a radius of 3000 m and a height of 6000 m;
- in a crosswind case and a windless situation of a three-dimensional version of a parallelepiped with lateral dimensions of 6000 × 6000 m and a height of 3000 m.

#### Compressible setting

The simulation takes place explicitly in the "classical" formulation of the problems of the outflow of a hot supersonic jet from a nozzle, in which the density of the mixture changes according to the law of an ideal gas. The atmosphere parameters (i.e., temperature, humidity, pressure, wind speed and their vertical profiles) are set in the form of tables or functional dependencies on the height in the initial and boundary conditions. Humid air is a mixture of gases and dry air, water vapor and combustion products. In this formulation, aerosol particles are specified without taking into account condensation and additional heat release, only for the purpose of determining the particle concentration profile for transfer to the setting of large-scale atmospheric currents.

#### Mathematical model of large-scale atmospheric currents

The mathematical model is based on the Navier–Stokes equations, the equation of energy, mass transfer, and written in variables relative to natural distributions of pressure, velocity, temperature and humidity. In this case, the assumption is made that artificial perturbations of the parameters of the medium are small in comparison with the natural parameters. The hydrostatic pressure component in the problem is significantly higher than the pressure changes that arise during the development of convective flows in the atmosphere. In the case of a *stationary atmosphere*, the velocities of the undisturbed atmosphere are equal to zero, while the density, temperature, and humidity depend, respectively, on the vertical coordinate *y*, i.e.,

$${\rho }_{0}=\rho \left(y\right), {T}_{0}=T\left(y\right),{C}_{0}=C\left(y\right),{P}_{0}=P\left(y\right).$$

The reactive jet and wind disturb the atmosphere velocities *w*, density, temperature, and pressure, which differ from the undisturbed ones. One may describe the atmosphere disturbed parameters as:

$$\rho ={\rho }^{\prime}+{\rho }_{0}; T=T\mathrm{^{\prime}}+{T}_{0}; P=P^{\prime}+{P}_{0}; C=C^{\prime}+{C}_{0}$$

where $\rho^{\prime}$, *T* $t{^{\prime}}$ and $C^{\prime}$ are the incremental values of the atmosphere density, temperature, pressure, and concentration, respectively.

The introduction of hygroscopic aerosol absorbing water vapor into the jet will lead to a decrease in vapor concentration and release of condensation heat.

Suppose that absorption occurs at the rate of diffusion of water vapor between particles on all particles with equal probability and the maximum amount of water condensed by each particle of mass *m*_*a*_ in time *t* is equal to *m*_*w*_ = *m*_*t*_—*m*_*a*_. At a concentration of hygroscopic aerosol in a jet equal to *n* (m^−3^), the average distance between particles is *d* = 1/*n*^1/3^ (m), the total amount of absorbed water is *M* = *n⋅m*_*w*_ (kg/m^3^). The particles absorb this mass of water in a diffusion time equal to $\tau =\frac{{d}^{2}}{D}=\frac{1}{D{n}^{\raisebox{1ex}{2}\!\left/ \!\raisebox{-1ex}{3}\right.}}$ (c)*,* where *D* – diffusion coefficient (m^2^/s), which determines the growth rate of particles due to the condensation of water vapor.

The water absorption rate per one aerosol particle with mass *m*_*a*_ will be equal to:

12 $${Q}_{w}=\frac{{m}_{w}}{\tau {d}^{3}}={m}_{w}\frac{n}{\tau }={m}_{w }D{n}^{\raisebox{1ex}{5}\!\left/ \!\raisebox{-1ex}{3}\right.}(\mathrm{kg}/\mathrm{s}\cdot {m}^{3}),$$

and will be limited by the amount of water in the air around the particle.

$${Q}_{w}={{m}_{w}^{\prime}D{n}^{\raisebox{1ex}{5}\!\left/ \!\raisebox{-1ex}{3}\right.}}$$, where $${{m}_{w}}^{\prime}=\mathrm{min}({m}_{w}; \frac{C{\rho }_{0}}{n}+{m}_{a })$$*.*

It should be noted that the case of evaporation from the surface of a hygroscopic particle is not considered and the rate of water absorption by a droplet is equal to:

$${Q}_{a}={Q}_{w}$$, if $${m}_{a }<{m}_{t}$$;

$${Q}_{a}=0$$, if $${m}_{a }\ge {m}_{t}$$.

The value of *Q*_*a*_ should be included in the equation for the water vapor concentration with the opposite sign (where is a sink of steam):

13 $$\frac{\partial {\rho }_{0}{C}^{\prime}}{\partial t}+{{\nabla}} \left({\rho }_{0}{\varvec{V}}{C}^{\prime}\right)={\nabla} \left(D{\nabla} {C}^{\prime}\right)-{V}_{y}\frac{\partial {\rho }_{0}{C}_{0}}{\partial y}-{Q}_{a}.$$

The term ${Q}_{a}E$ will be added to the energy equation:

14 $$\frac{\partial {\rho }_{0}\mathrm{C^{\prime}}}{\partial t}+{\nabla} {\rho }_{0}\mathrm{C^{\prime}}{\varvec{V}}=-{\rho }_{0}{{\varvec{V}}}_{y}{\mathrm{C}}_{p}(\frac{\partial {T}_{0}}{\partial y}-\frac{\rho ^{\prime}}{{\rho }_{0}}\frac{\partial {T}_{0}}{\partial y}+g)+{\nabla} ({\lambda }_{t}{\nabla} (T^{\prime}+{T}_{0}))+Q+{Q}_{a}E$$

During the absorption of water by an aerosol particle (${Q}_{a}>0$), energy will be released, and during evaporation, it will be absorbed (absorption is not taken into account in this model).

The change in the mass of aerosol particles per unit volume per unit time divided by the number of particles ($\frac{{dM}_{a}}{dt}$, kg/(s⋅m^3^), is described by the equation:

15 $$\frac{d{M}_{a}}{dt}={D}_{user}\cdot {m}_{a}+{F}_{user},$$

where ${D}_{user}$ and ${F}_{user}$—numeric constants set via the interface.

Substituting the water absorption rate ${Q}_{a}$, we get:

$$\frac{d{M}_{a}}{dt}={{(m}_{t}}^{\prime}-{m}_{a })\mathrm{D}{n}^\frac{5}{3}, {\text{where }}{{ m}_{t}}^{\prime}=\mathrm{min}({m}_{t}; \frac{C{\rho }_{0}}{n}+{m}_{a }).$$

It means that ${D}_{user}= -\mathrm{D}{n}^\frac{5}{3}$ and ${F}_{user }={{m}_{t}}^{\prime}\mathrm{D}{n}^\frac{5}{3}$.

With this in mind, the following system of equations is solved in the FlowVision:

1. Continuity equation:
  16 $$\frac{\partial \rho ^{\prime}}{\partial t}+{\nabla} \left({\rho }_{0}V\right)=0;$$
2. Navier-Stocks equation:
  17 $$\frac{\partial {\uprho }_{0}V}{\partial t}+{\nabla} \left({\rho }_{0}V\otimes V\right)=-{\nabla} p+{\nabla} \cdot \widehat{\tau }-\rho ^{\prime}g$$
3. Energy equation:
  18 $$\frac{\partial {\rho }_{0}h^{\prime}}{\partial t}+{\nabla} {\rho }_{0}h{^{\prime}}V=-{\rho }_{0}{V}_{y}{\mathrm{C}}_{p}(\frac{\partial {T}_{0}}{\partial y}-\frac{\rho {^{\prime}}}{{\rho }_{0}}\frac{\partial {T}_{0}}{\partial y}+g)+{\nabla} ({\lambda }_{t}{\nabla} (T{^{\prime}}+{T}_{0}))+Q+{Q}_{a}E$$
4. Equation of vapor mass transfer:
  19 $$\frac{\partial {\rho }_{0}C{^{\prime}}}{\partial t}+{\nabla} \left({\rho }_{0}VC{^{\prime}}\right)={\nabla} \left(D{\nabla} C{^{\prime}}\right)-{V}_{y}\frac{\partial {\rho }_{0}{C}_{0}}{\partial y}-{Q}_{a}.$$
5. Equation of aerosol particle mass transfer:
  20 $$\frac{d{M}_{a}}{dt}={{(m}_{t}}^{{\prime}}-{m}_{a })\mathrm{D}{n}^\frac{5}{3}, \mathrm{where}{{ m}_{t}}^{{\prime}}=\mathrm{min}({m}_{t}; \frac{C{\rho }_{0}}{n}+{m}_{a }).$$
  where *V* is the velocity vector (m/s); $\widehat{\tau }$ is the viscous stress tensor (Pa); *g* is the free-fall acceleration vector (m/s^2^); *h* = *C*_*p*_*T* is the enthalpy (it is assumed that heat capacity is constant); λ_*t*_ is the coefficient of thermal conductivity (the sum of molecular and turbulent thermal conductivity); *Q* and *Q*_*a*_* E* are the energy sources; *C* is the mass fraction of water vapor; *m*_*a*_ is the mass of one particle; *m*_*t*_ is the maximum amount of water absorbed by one particle; *D* is the diffusion coefficient; *d* = 1/*n* ^(1/3)^ is the average distance between particles; *n* is the concentration of absorbing particles in 1 m^3^.

For the equation of motion of particles it is necessary to set lifting force (only along vertical axis Y):

21 $${F}_{d}=-\mathrm{9,81}\cdot \left(1-\frac{{\rho }^{\prime}}{{\rho }_{d}}\right),$$

where *ρ*_*d*_ is the particle density, which increases as moisture condenses on a particle of constant diameter.

For increments *T'* <  < *T*_*0*_, the relative density of the mixture ρ' is calculated as follows:

If *C* < *C*_*max*_ : $$\rho {^{\prime}}=-{\rho }_{0}\frac{{T}^{{\prime}}}{{T}_{0}}+{\rho }_{0}(1-\frac{{\mu }_{air}}{{\mu }_{w}}){C}^{{\prime}}$$;

If *C* ≥ *C*_*max*_ : ${\rho }^{{\prime}}=-{\rho }_{0}\frac{{T}^{{\prime}}}{{T}_{0}}+{\rho }_{0}\left(1-\frac{{\mu }_{air}}{{\mu }_{w}}\right)\left({C}_{max}-{C}_{0}\right)+{\rho }_{0}\left(C-{C}_{max}\right).$ where *C*_*max*_ is the maximum vapor content, at *C* > *C*_*max*_ there is condensation and phase transition energy release, equal to *q* = 2.26 MJ/kg. As temperature and/or water vapor concentration increases, air density decreases—i.e. warmer and wetter air is lighter than cooler or drier air. But when *C*_*max*_ is reached, moisture begins to precipitate in the form of droplets, and air density begins to increase because the water droplets take up almost no volume, and the partial pressure of water vapor does not change, μ_*w*_ and μ_*air*_ are the molecular weight of water vapor and the average molecular weight of air, 18 and 29, respectively.

The source of energy due to condensation Q, given in the energy equation, is determined by:

$$Q=\rho q\frac{\partial C}{\partial t} at C > {C}_{max};$$

22 $$Q = 0 at C<{C}_{max}.$$

In addition to this system of equations, a system of equations of *k-ε* turbulence model is solved, in which the turbulent viscosity and turbulent thermal conductivity are determined.

Attention should be paid to the right part of the energy equation (the first composite term with the atmospheric temperature gradient). The first term contains the generation of enthalpy variation ("heat") due to the air moving upward. Of course, there is no heat generation—rising causes an increase in relative temperature—this is the term with the multiplier $( \frac{T{^{\prime}}}{{T}_{0}}-1)$. There is also cooling due to the work of pressure forces—this is the term with multiplier -*g*. If there are several substances (air and combustion products), additionally the mass transfer equation of each component is solved.

### The problem is solved in an axis symmetric setting for options without wind and in a full 3D setting for options with wind. The boundary conditions (BC) are shown in Fig. 1

**Figure 1:**
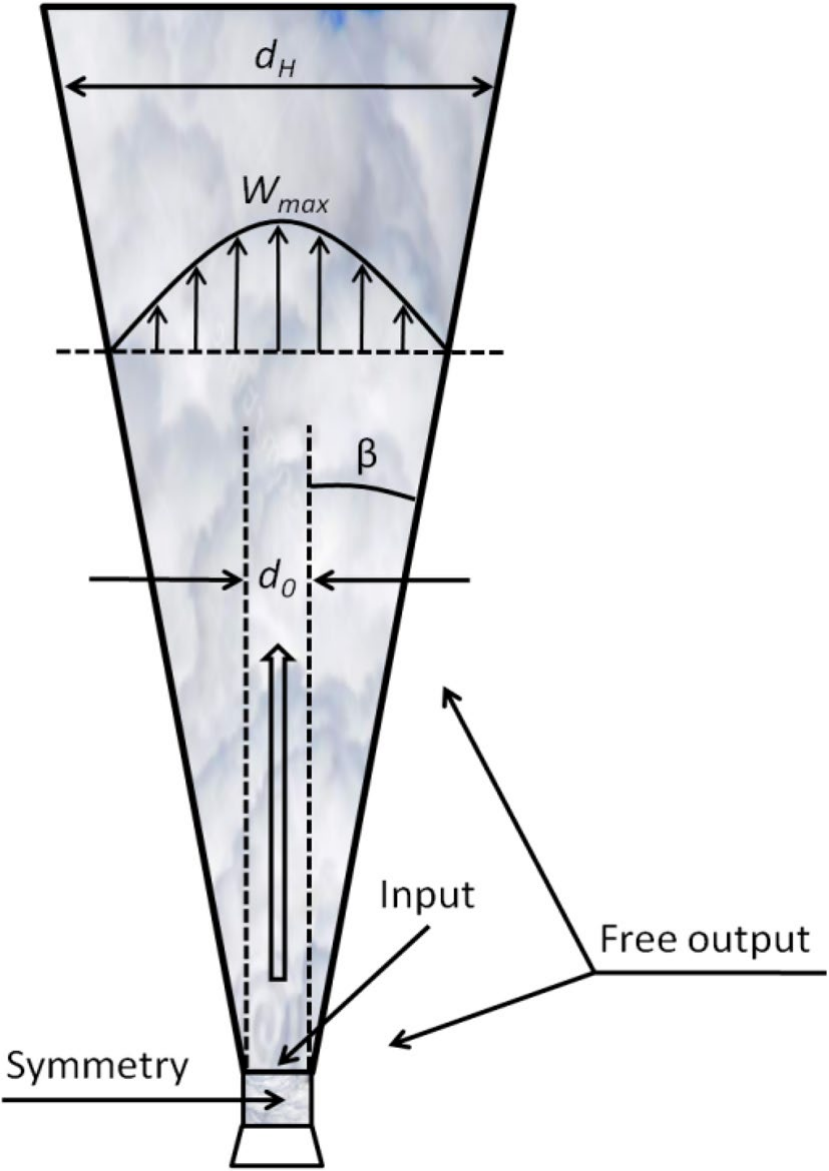
Jet shape and boundary conditions.

The arrangement of the BC for setting large-scale atmospheric currents is similar to the arrangement in the compressible setting, except for the BC "on the ground", where the data obtained in the compressible statement is set. i.e.; data from the compressible statement are presented in the form of functional dependencies on the radius and are set on the BC for large scales. In the design modes, where the wind speed is set, this speed is also considered for the initial conditions.

#### Computational grid

**Figure 2:**
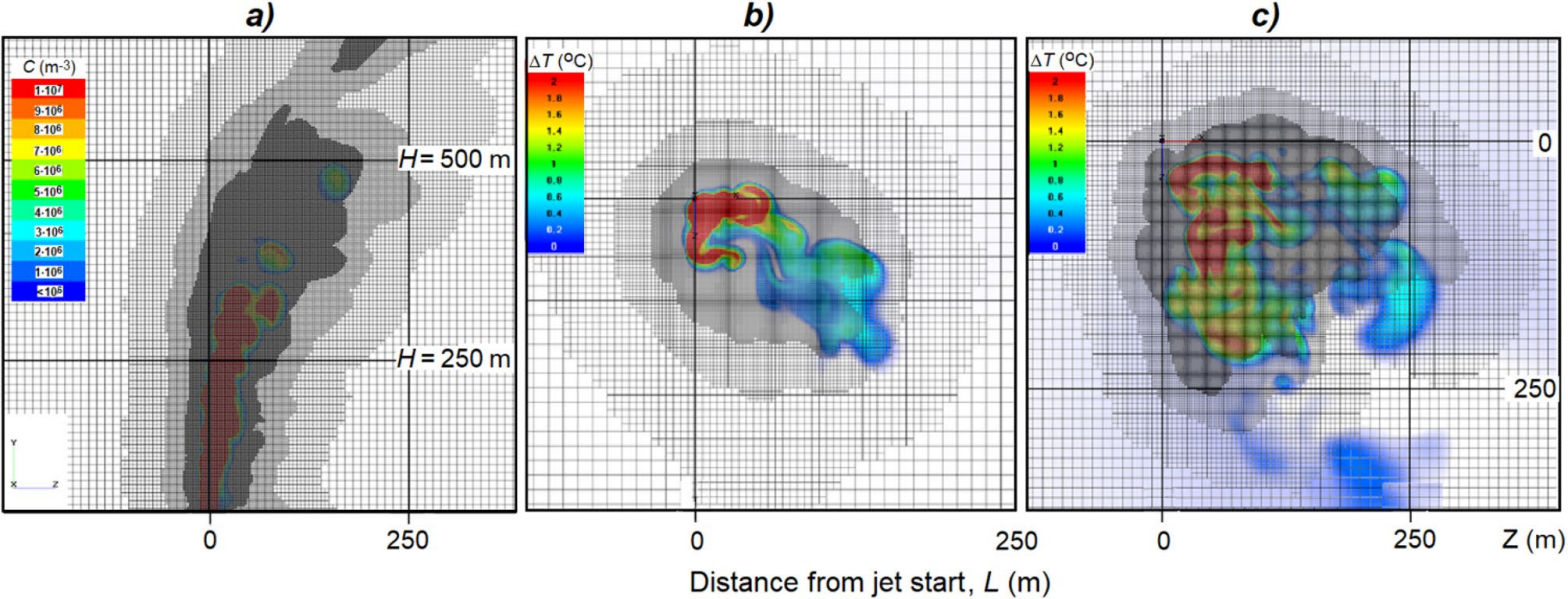
Computational grids (option with wind): vertical cross-section (**a**) of the aerosol concentration field (*C*, m^-3^); and horizontal cross-sections of the temperature excess fields (Δ*T*, ^o^C) at altitudes *H* = 250 (**b**) and 450 m (**c**) from the jet start level.

An irregular mesh was used to calculate the parameters of the compressible flow, which is condensed in the region of the main jet (Fig. 2a). The mesh adapts in the jet outlet area up to adaptation level 3. The minimum cell size of the computational grid with its adaptation is 0.04 m, and the number of computational cells is 37 thousand.

Similarly, an irregular grid was also used to calculate the parameters of large-scale atmospheric currents that are condensed in the area of the main jet (Fig. 2b,c). The minimum starting cell size is 10 m for this case. Furthermore, the grid adapts in the area of the greatest disturbances up to the 3^rd^ adaptation level. The adaptation of the 3^rd^ level is volumetric; it is performed according to the condition of the particles concentration of the dispersed phase. The total number of calculation cells is about 90 thousand for setting without wind and up to 8 million calculation cells for setting with wind.

Observe that the *integration step* was specified using the Courant-Friedrichs-Lewy (CFL) number.

When calculating the parameters of the *compressible jet* of the engine, the *time step* was chosen to be CFL = 25, which approximately corresponds to 0.002 s.

When calculating the parameters of *atmospheric currents* at a *distance from the engine*, the time step chosen to be CFL = 25, which approximately corresponds to 0.5 s.

In addition, in both mathematical problems, the time step constraint CFL_diffuse_ = 1 was used. Integration scheme: implicit 2^nd^ order of accuracy.

#### Model verification

The solution of the proposed mathematical model was carried out as follows:

### (A) Adiabatic rise of heated air in a vertical "pipe"

The vertical air rise in the computational domain with a height of 6 km without friction at a constant speed was considered. At the inlet to the pipe, a speed of 1 m/s and a temperature of 10 °C are set, at the outlet at an altitude of 6 km – a free exit with an overpressure relative to the pressure at a given height is set. For comparison, the formula used for the change in temperature with height in the adiabatic process: ${T}_{adiabat}\left(Y\right)={T}_{0}{\cdot (\frac{p\left(Y\right)}{{p}_{0}})}^{1-1/k}$.

**Figure 3:**
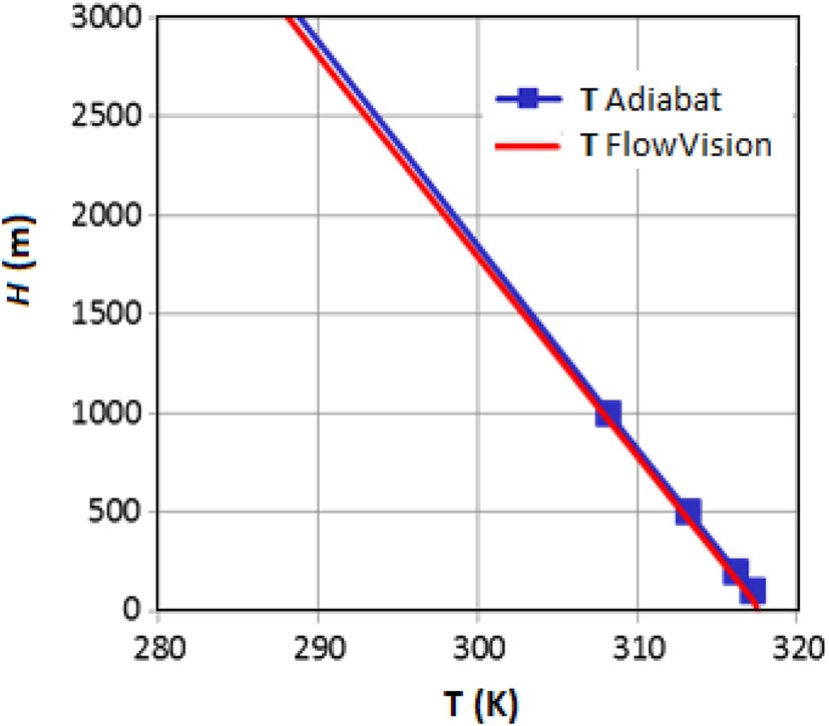
Comparison of temperature graphs for adiabatic rise and in FlowVision.

From Fig. 3 it follows that the FlowVision solution shows an agreement with the analytical dependence within 0.1% up to an altitude of at least 3 km.

### (B) Simulation of a vertical heated jet in the atmosphere up to a height of 100 m

**Figure 4:**
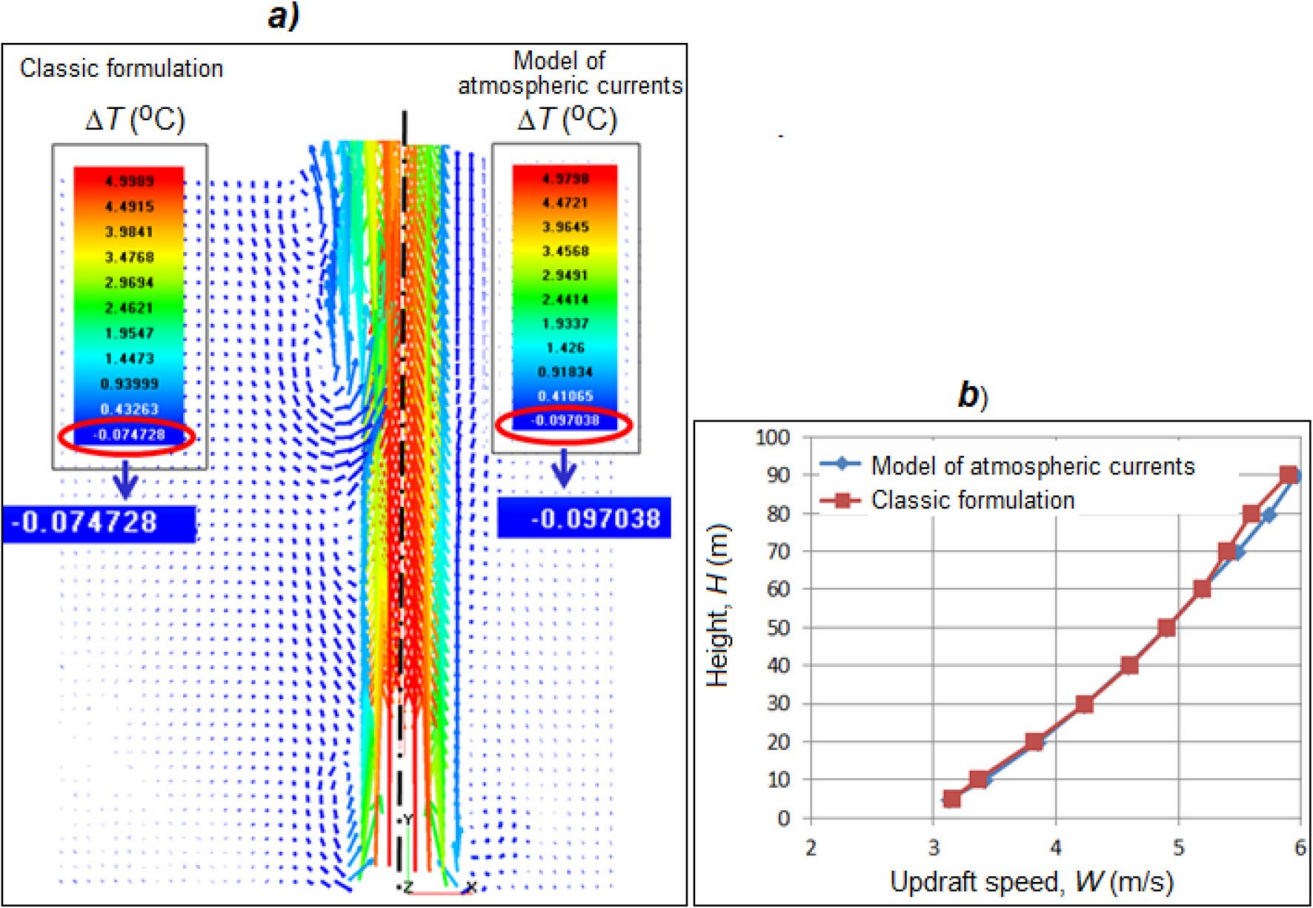
Comparison of the flow patterns in the classical formulation and in the FlowVision atmospheric current model: (**a**) the velocity field with the coloring of the vectors for the temperature rise Δ*T* (°C); (**b**) dependence of the updraft speed *W* (m/s) on the height.

In this case, the classical approach to modeling and the large-scale FlowVision model of atmospheric currents are compared using the example of a vertical air jet entering the atmosphere at a speed of 3 m/s and an excess temperature of 5 °C. For each computational case, the same computational grid is constructed using a uniform cubic one with a side of 20 m. The step in the integration time is 1.5 s (constant). The results have shown that the jet profiles and the flow pattern are in good agreement with each other (Fig. 4).

In the classical formulation, more vortex formation is observed, which can be associated with the presence of transverse density gradients. In both cases, a negative excess temperature (from − 0.03 to − 0.09 °C) is obtained due to the ejection effect, where the lower layers, which are colder than the core of the jet, are carried upward by the jet and cooled during expansion.

**Figure 5:**
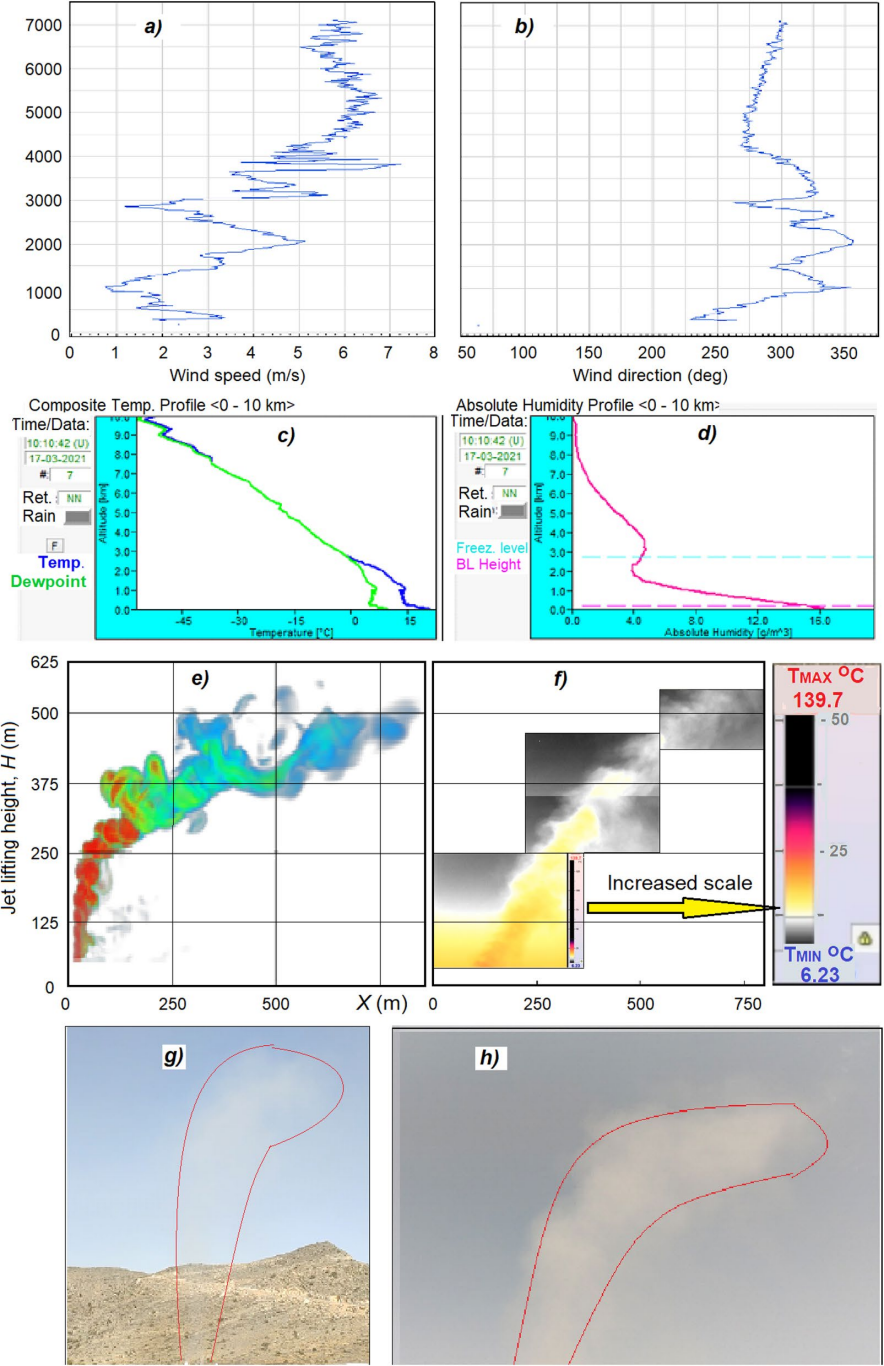
Comparison of the simulated and observed jet characteristics (without accounting of condensation heat), initiated at an altitude of 1595 m above sea level during trial field experiment on Jebel Jais Mountain of UAE on March 17, 2021. Wind speed (**a**) and direction (**b**) measured by Lidar Halo Photonics Streamline XR; temperature (**c**) and specific humidity (**d**) profiles measured by microwave radiometer RPG HATPRO-G4; Simulated jet shape (**e**); combined thermal images of jet (**f**) measured by infrared scanning thermograph IRTIS-2000C at 3 viewing angles; Visual images by GoPro Hero-9 Camera (**g**,**h**).

The correctness of the theoretical model and numerical results to predict real artificial buoyancy jets was confirmed in the first approximation through case by case comparison with experimental data. Here Fig. 5 shows results of the first field experiment in UAE on Jebel Jais Mountain on March 17, 2021. Equipment was mounted on altitude of 1595 m above sea level. Vertical wind profile measured by Lidar Halo Photonics Streamline XR (Fig. 5a,b), temperature lapse rate and specific humidity profile measured by Microwave radiometer RPG HATPRO-G4 (Fig. 5c,d) were used as initial data of the model. Thermal images of buoyancy jet were measured by infrared scanning thermograph IRTIS-2000C at 3 viewing angles of the sky (Fig. 5f), while visual shape of jet was captured by GoPro Hero-9 Camera (Fig. 5g,h). Simulated jet is represented in Fig. 5e. Ground Weather Station Vaisala WXT-536 gave local temperature, wind, humidity and solar flux on the ground level. The temperature lapse rate according to Abu Dhabi airport sounding data for 12 h UTC in the height layer from 1493 to 2519 m was equal to γ = 7.4 °C/km, and air humidity was 19–21%. From Fig. 5 it follows that simulated and observed characteristics of the jet (lift height and shape) are in a good agreement with each other, though more comparisons are needed to validate implemented model.

## Results

### Buoyancy jet simulation

After adaptation and testing of the FlowVision software, numerical experiments were carried out to study the parameters of updrafts initiated by a vertically directed reactive jet saturated with a coarsely dispersed hygroscopic aerosol. Three-dimensional fields of aerosol concentration, temperature, and jet velocity were considered depending on the vertical profiles of temperature, humidity and wind speed in the atmosphere.

In the numerical implementation of the model, it is assumed that the total buoyancy jet power ${P}_{\Sigma }$ is the sum of the jet power ${P}_{J}$ and the power of the condensation heat source ${P}_{C}$:

$${P}_{\Sigma }= {P}_{J}+{P}_{C}.$$

**Figure 6:**
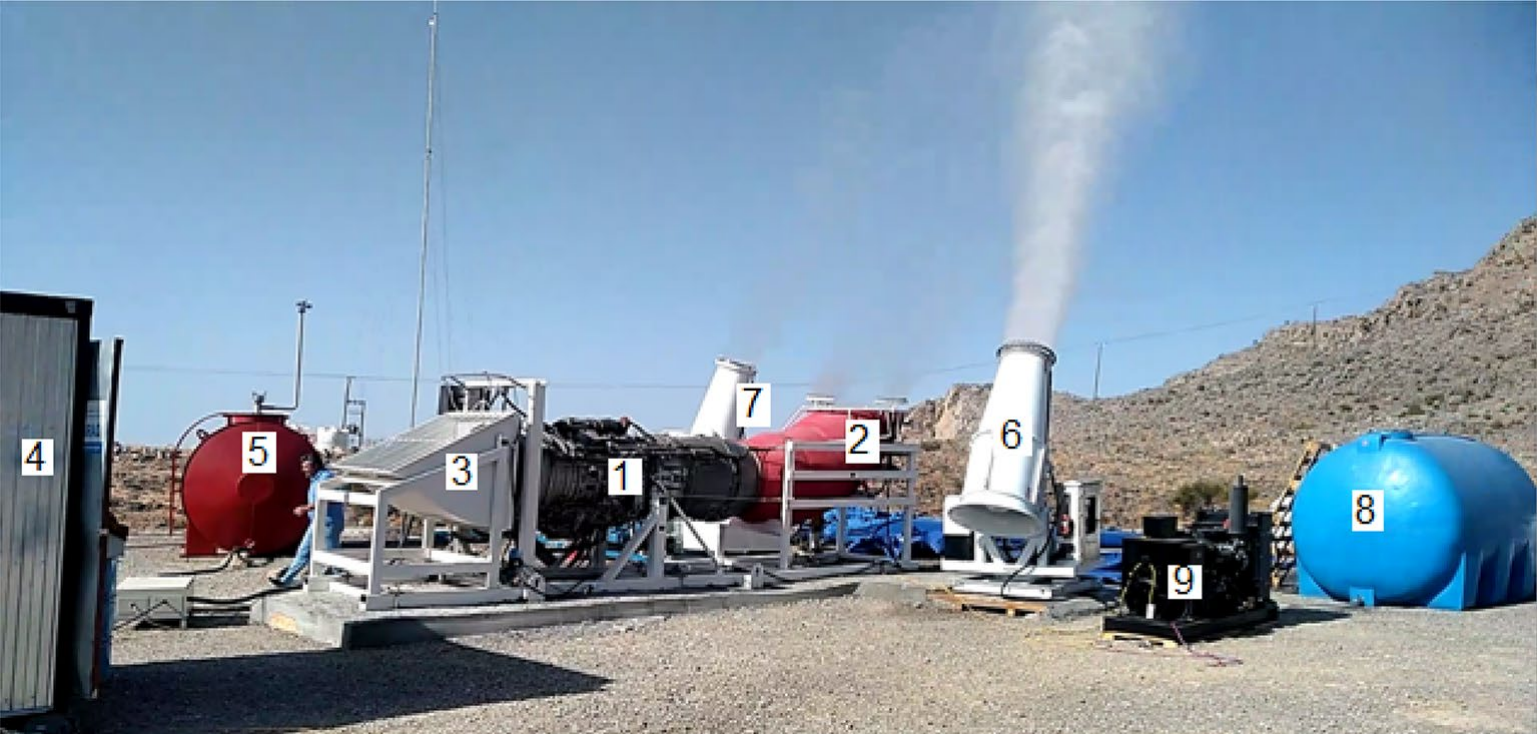
Experimental complex in operation: (1) aircraft engine D-30; (2) nozzle for directing the jet up; (3) engine air receiver; (4) engine start and control cabinet; (5) jet fuel tank; (6) Fog Cannon JY-60; (7) Fog Cannon WP-60; (8) tank for hygroscopic substances solutions; (9) diesel generator 3 × 380 50 kW to feed the Fog Cannon JY-60.

The value of ${P}_{J}$ is maximum at the moment of the jet outflow from the jet engine (Fig. 6) and decreases as it rises, and the ${P}_{C}$ source is switched on when the relative humidity of the air in the jet exceeds the hygroscopic point of the injected aerosol substance. The parameters of both sources are shown in Tables 1 and 2.

**Table 1:** Parameters of the jet initiated by the experimental device.

No	Parameter	Designation	Measure unit	Value
1	Initial jet temperature rise over environment	*T*_*0*_	^o^C	300
2	Initial jet speed	*W*_*0*_	m/sec	≈ 300
3	Initial jet mass flow	*M*_*0*_	kg/sec	≈ 200
4	Initial jet diameter on start	*D*_*0*_	m	1.2
5	Jet power	*P*_*J*_	MW	69
6	Operating time in 1 experiment	*t*	Min	20

**Table 2:** Parameters of hygroscopic aerosol stream on the output of Fog Cannon.

No	Parameter	Designation	Measure unit	Value
1	Mass of sprayed aqueous solution	*M*_*s*_	kg/hour	3600
2	Air flow at the outlet of Fog Cannon	*M*_*o*_*’*	m^3^/sec	14
3	Aerosol jet lift at no wind	*H’*	m	≈ 40
4	Nozzles number	*N*	Pcs	80
5	Size of spraying droplets	*d*_*s*_	μm	≈ 15
6	Initial size of formed aerosol particles	*d*_*a*_	μm	≈ 10
7	Number of formed aerosol particles	*N*	Pcs./sec	4.83·10^11^
9	Humidity at which condensation begins: on CaCl_2_ aerosols particles on carbamide aerosols particles on NaCl aerosols particles	*f*	%	6 49 75.3
10	Heat output of water vapor condensation: on CaCl_2_ aerosols particles on carbamide aerosols particles on NaCl aerosols particles	*P*_*c*_	MW	2.5 7.2 19.5

Numerical experiments were carried out on the supercomputer Tornado of the South Ural State University Intel Xeon-X5680 with a total number of computing processors 480/960 cores, each had 3.3 GHz and 24 GB of RAM. The calculation time of 1 h of physical time (~ 8 million cells) is 20 h (with optimal computer performance).

The results of the numerical experiments performed according to the jet parameters and aerosol generators as specified in Tables 1 and 2.

**Figure 7:**
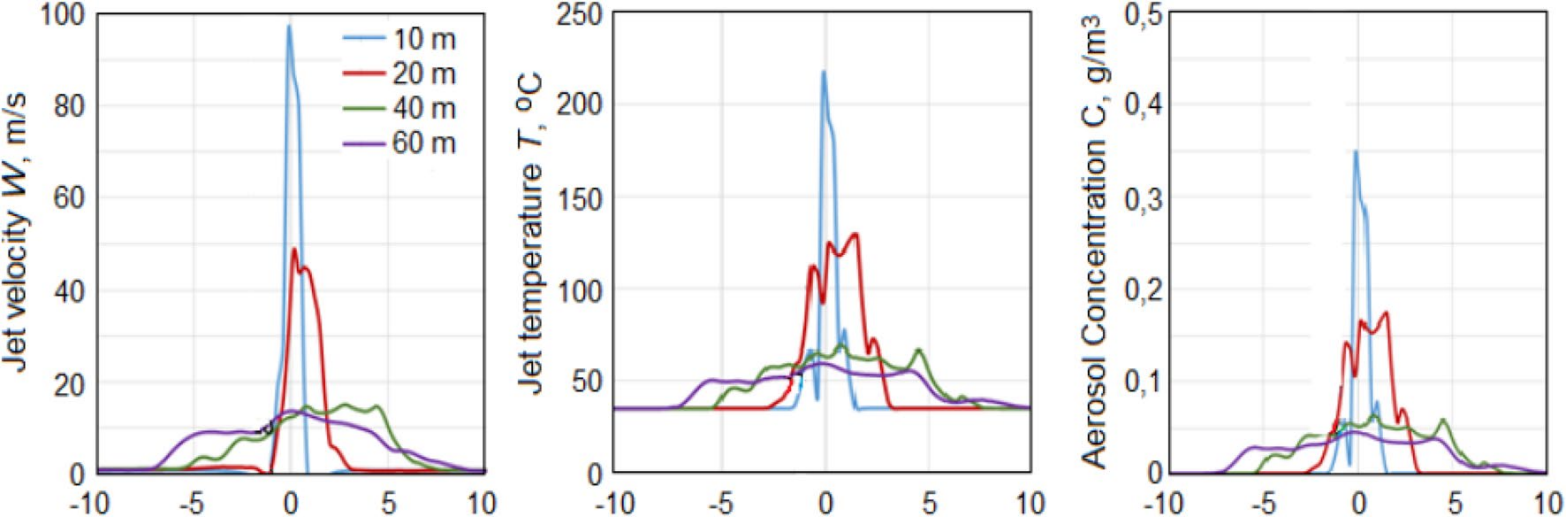
Instantaneous patterns of radial distributions of the updraft speed *W* (m/s), temperature *T* (°C) and aerosol concentration *C* (g/m^3^) at altitudes of 10, 20, 40 and 60 m in a windless atmosphere at γ = 6.5 °C/km and *f* = 45% 20 s after the launch of the turbojet device shown in Fig. 5.

**Figure 8:**
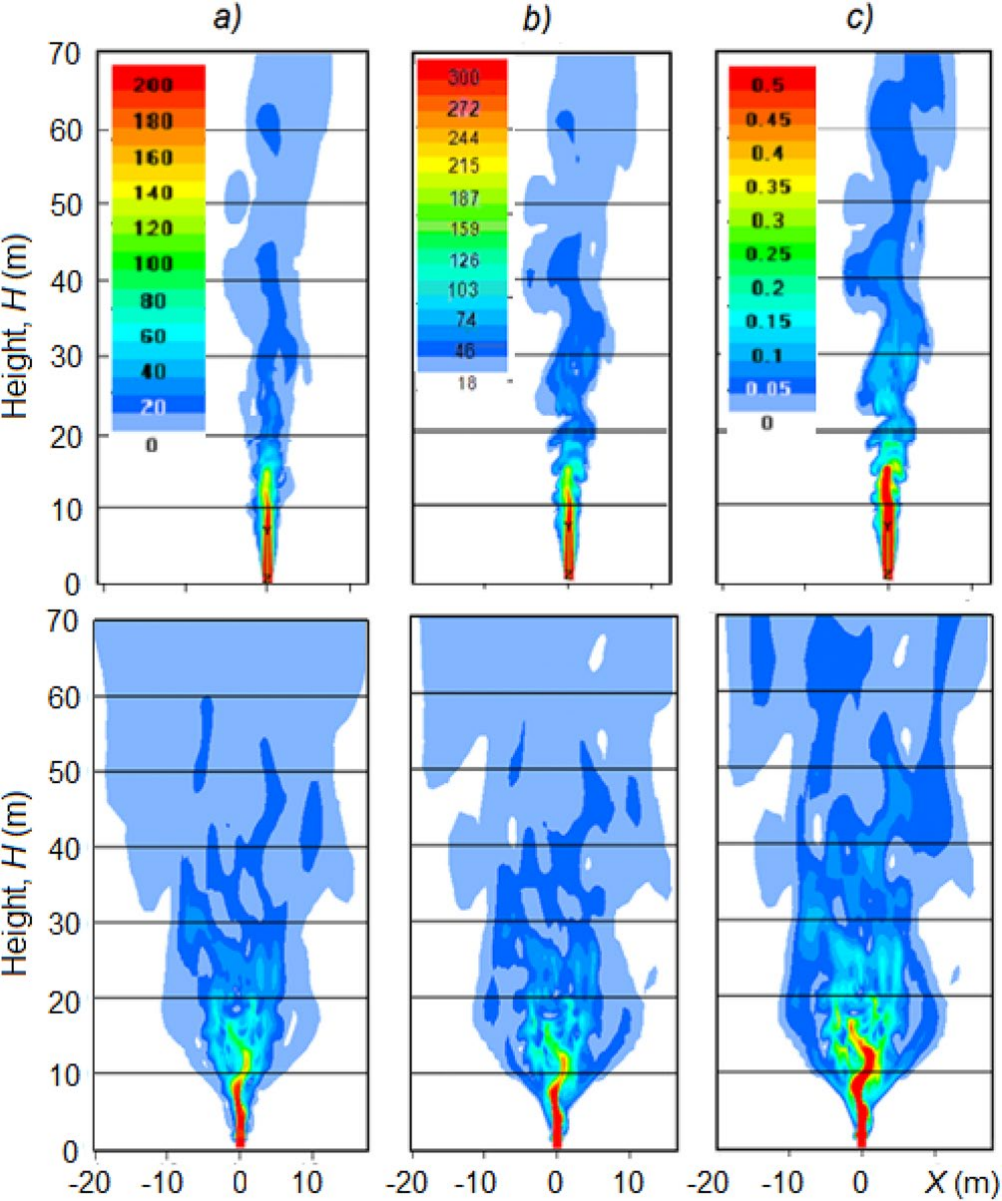
Vertical section of: (**a**) jet velocity *W* (m/s), (**b**) temperature *T* (^o^C) (**b**), and (**c**) aerosol concentration *C* (g/m^3^) in windless atmosphere (*U* = 0 m/s) without inversion layers and γ = 6.5 °C/km, *f* = 45%. Top row without spraying of hygroscopic solutions, bottom row with spraying of solutions.

**Figure 9:**
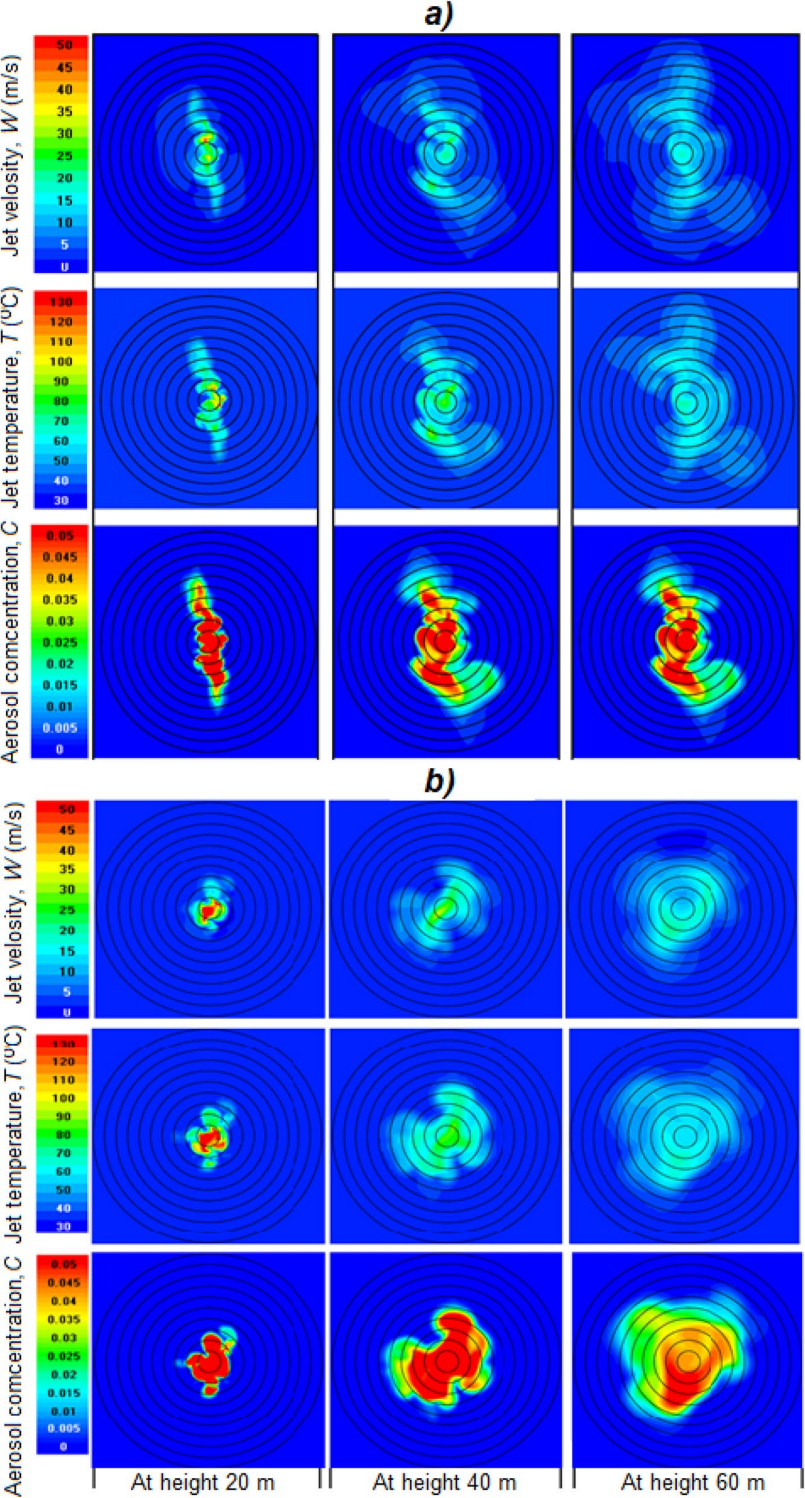
Horizontal sections of *W*, *T* and *C* fields at altitudes of 20, 40, 60 m in windless atmosphere with γ = 6.5 °C/km and *f* = 45% at 25 s (**a**) and 28 s (**b**) of simulation.

**Figure 10:**
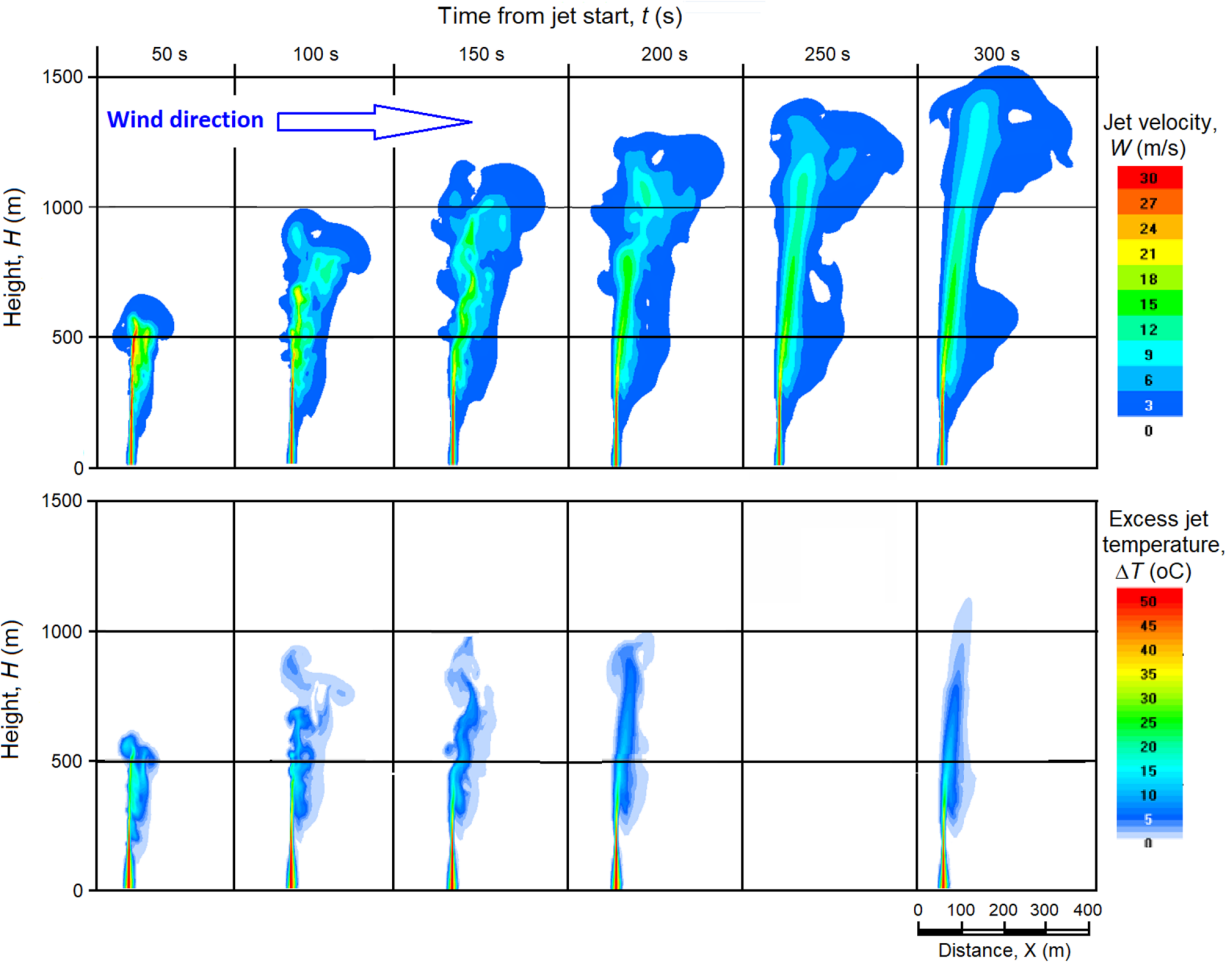
Vertical sections of *W* (m/s) (on the top) and Δ*T* (°C) (on the bottom) fields at wind speed at all heights *U* = 1 m/s in 50, 100, 150… 300 s after the start of engine operation.

**Figure 11:**
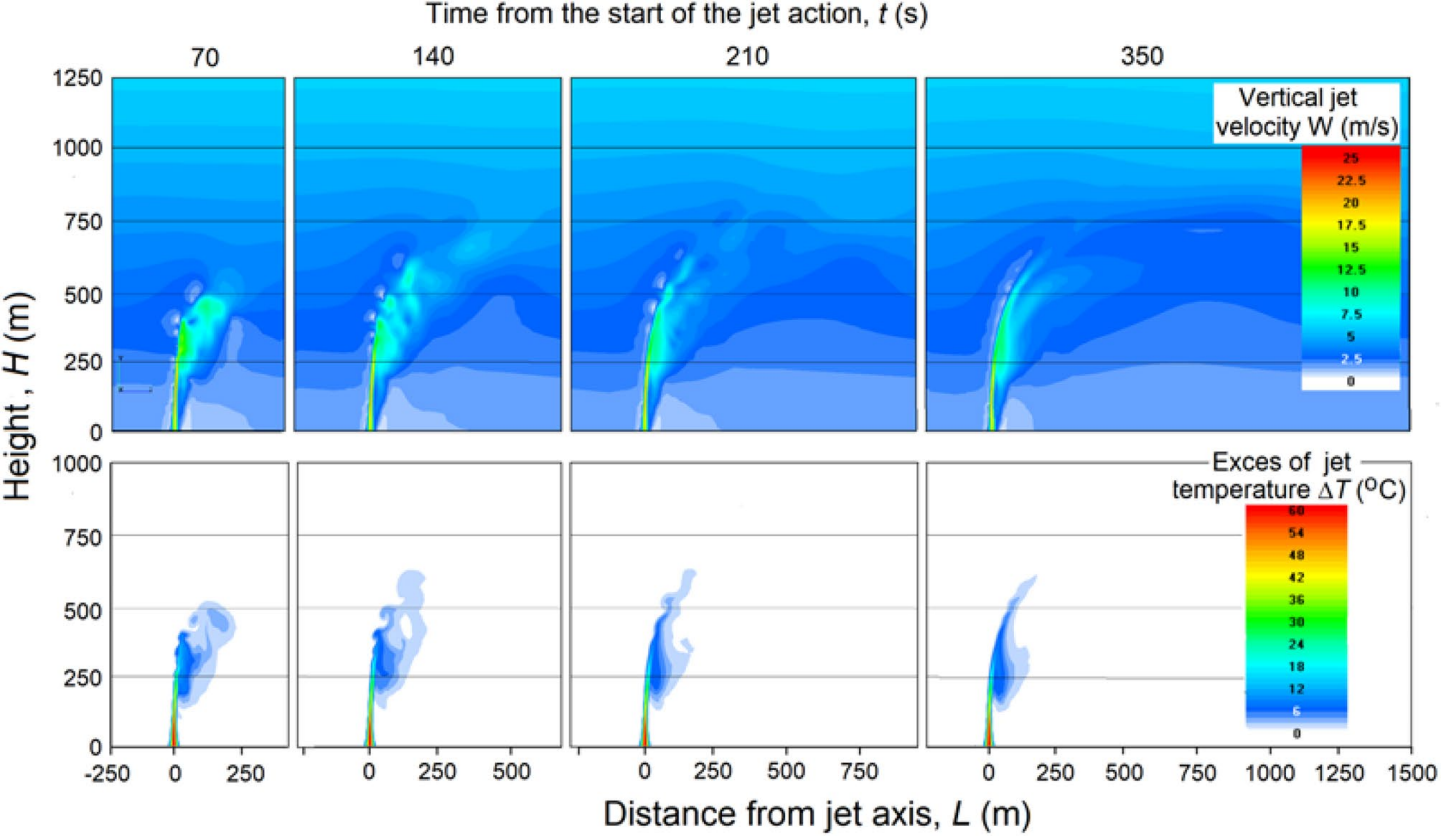
Vertical sections of *W* and Δ*T* fields with wind speed profile *U* = 1 + 0.004*H*, γ = 6.5 °C/km, *f* = 45%.

**Figure 12:**
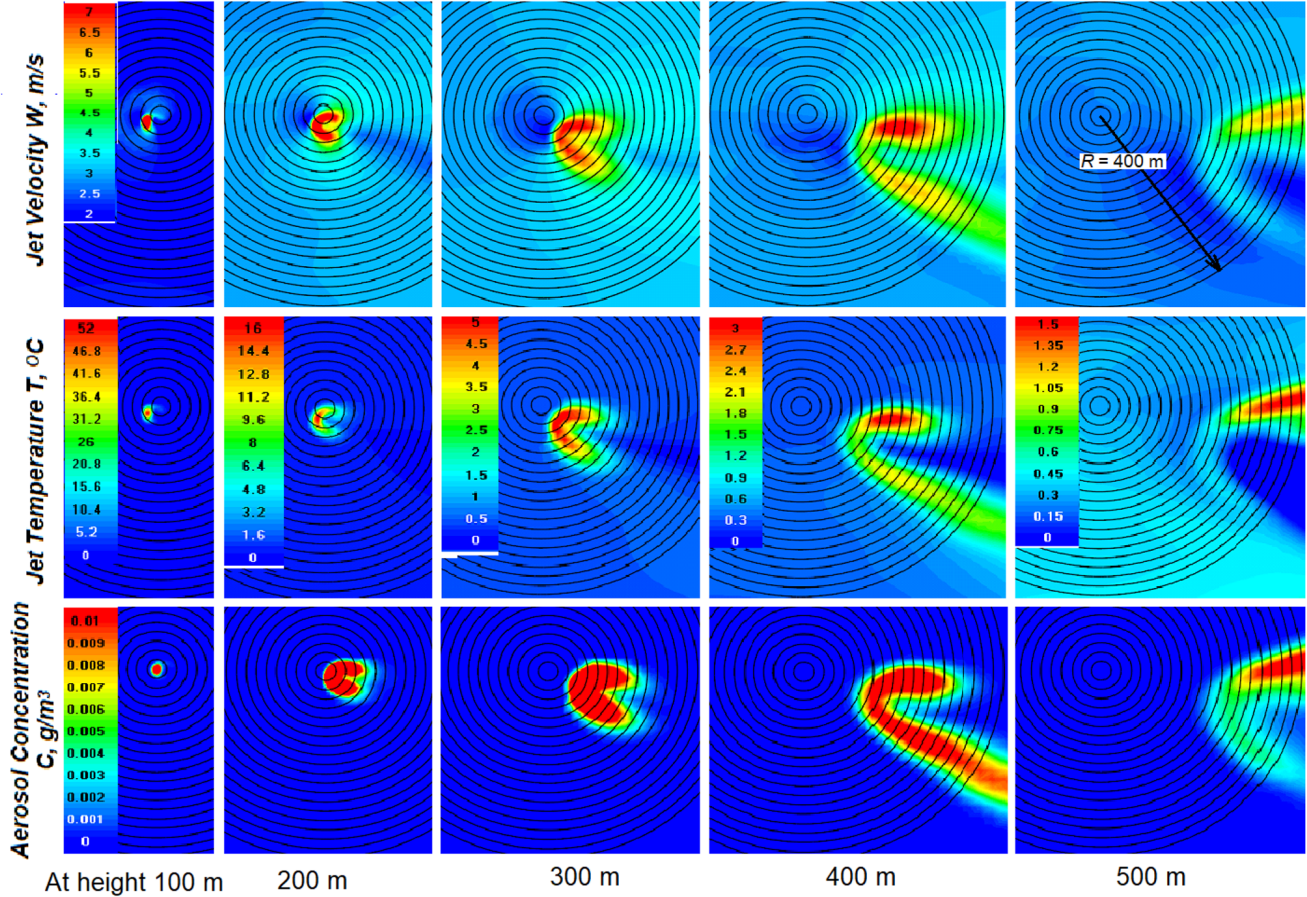
Horizontal sections of the *W*, *T* and *C* fields at the indicated heights in the presence of wind *U* = 1 m/s, γ = 6.5 °C/km and *f* = 45%.

**Figure 13:**
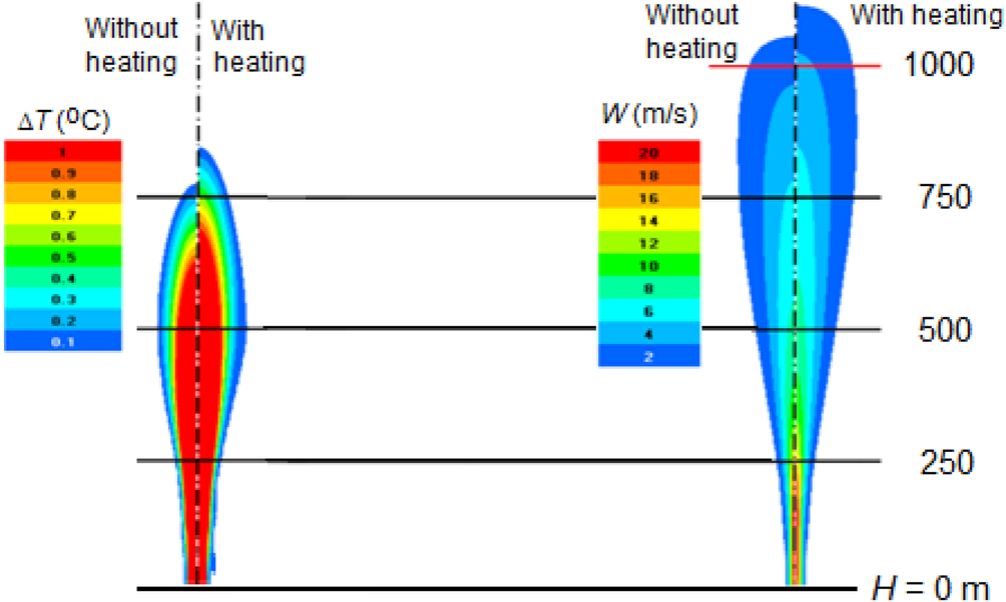
Fields of the jet temperature rise Δ*T* (^o^C) and updraft velocity *W* (m/s) with and without feeding *P*_*C*_ = 5.14 MW energy in a windless atmosphere at γ = 6.5 °C/km.

**Figure 14:**
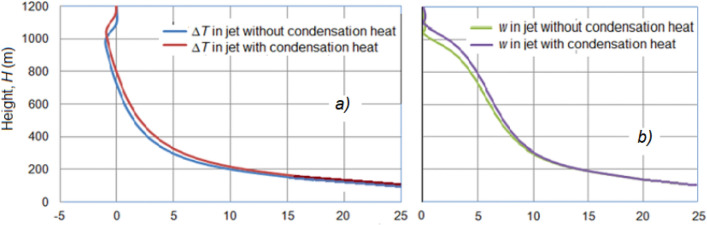
Vertical profiles of temperature excess Δ*T* (°C) (**a**) and updraft speed *W* (m/sec) (**b**) in a jet with feeding *P*_*C*_ = 5.14 MW and without feeding in a windless atmosphere at γ = 6.5 °C/km.

**Figure 15:**
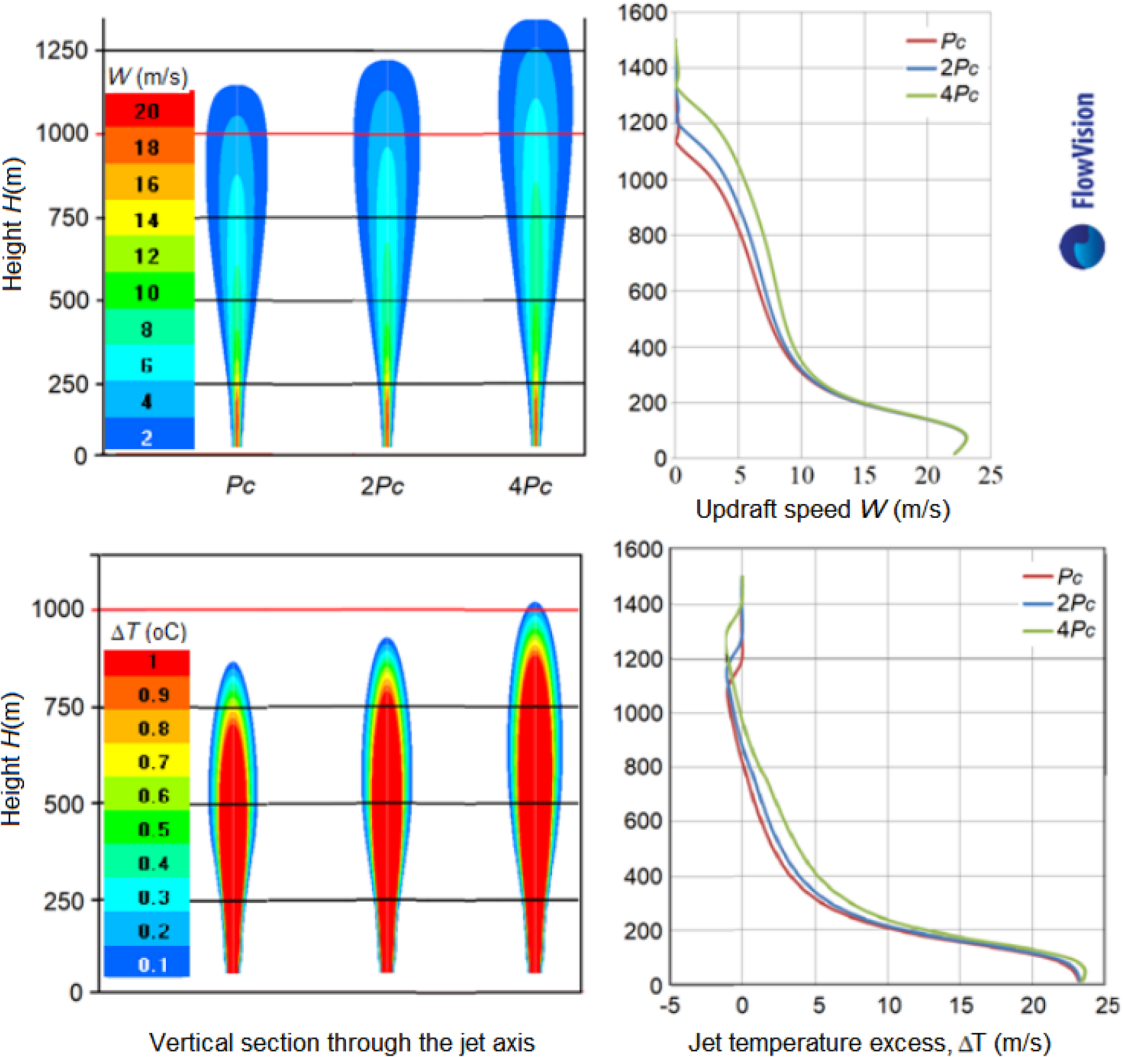
Vertical sections of *W* and Δ*T* at jet feeding *P*_*C*_, 2*P*_*C*_ and 4*P*_*C*_ in a windless atmosphere and γ = 6.5 °C/km.

**Figure 16:**
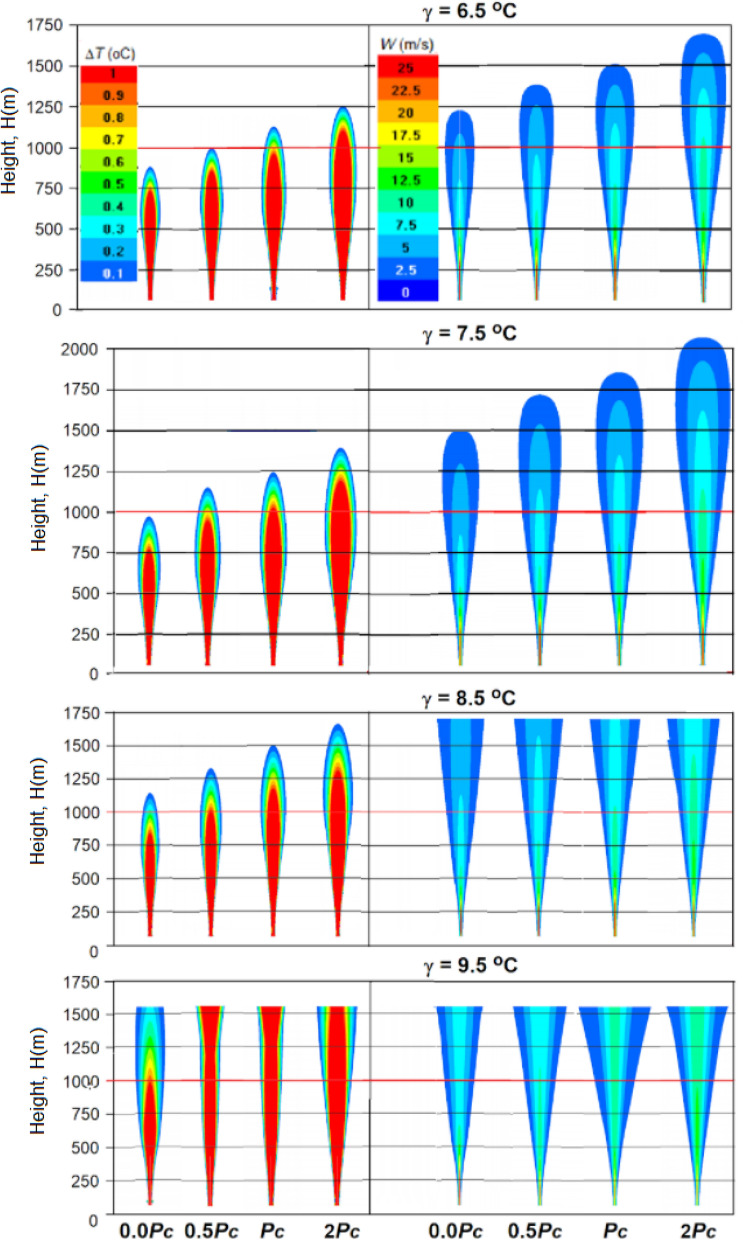
Vertical sections of Δ*T* and *W* fields for the jet with feeding *P*_*C*_ = 151 MW at γ = 6.5, 7.5, 8.5 and 9.5 °C/km in a windless atmosphere with *f* = 70%*.*

**Figure 17:**
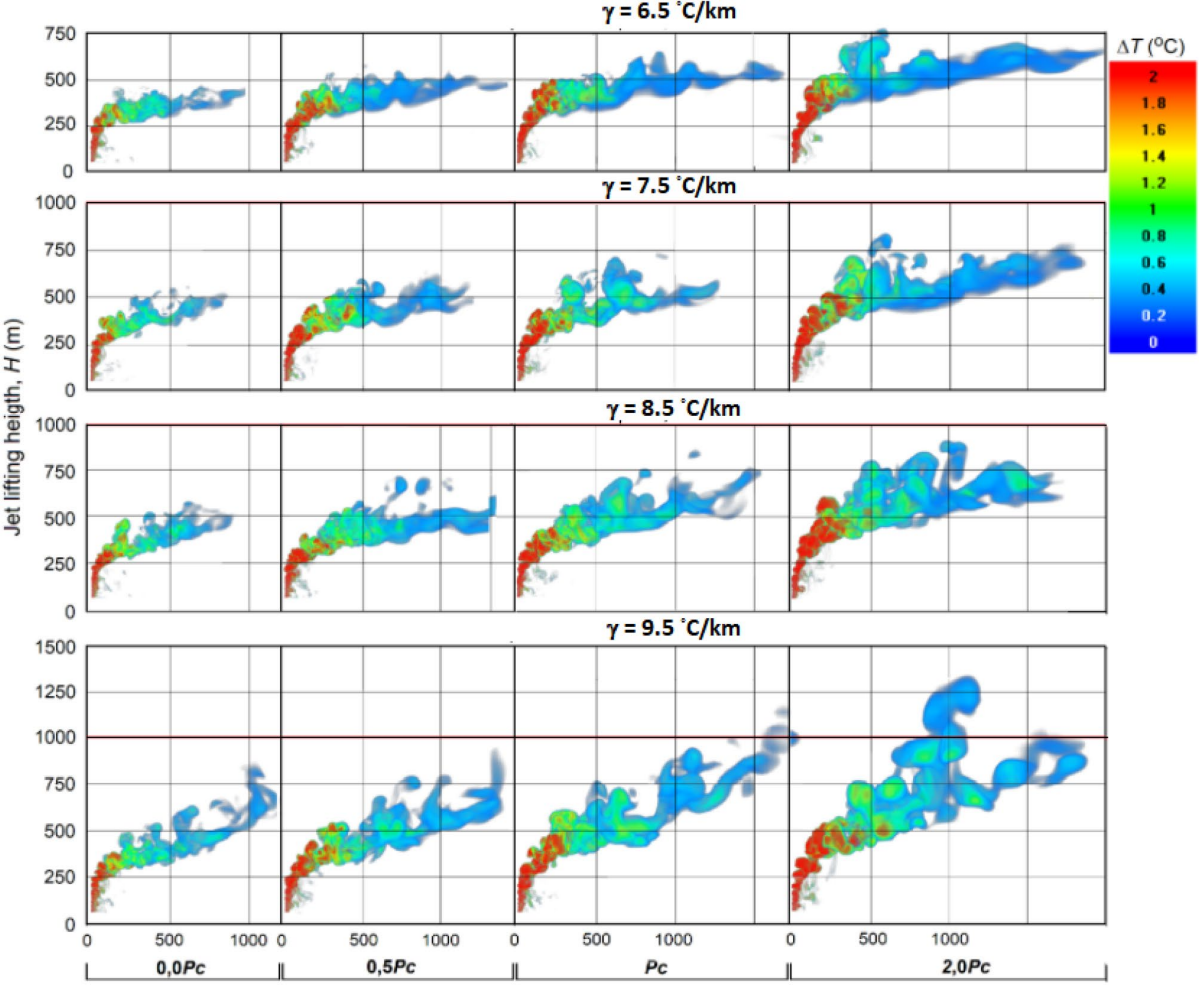
Vertical sections of excess temperature Δ*T* (^o^C) at feeding *P*_*C*_ = 151 MW, vertical wind profile *U* = 1 + 0.005*H* m/s, *f* = 70%, different temperature lapse rates γ = 6.5, 7.5, 8.5 and 9.5 °C/km.

**Figure 18:**
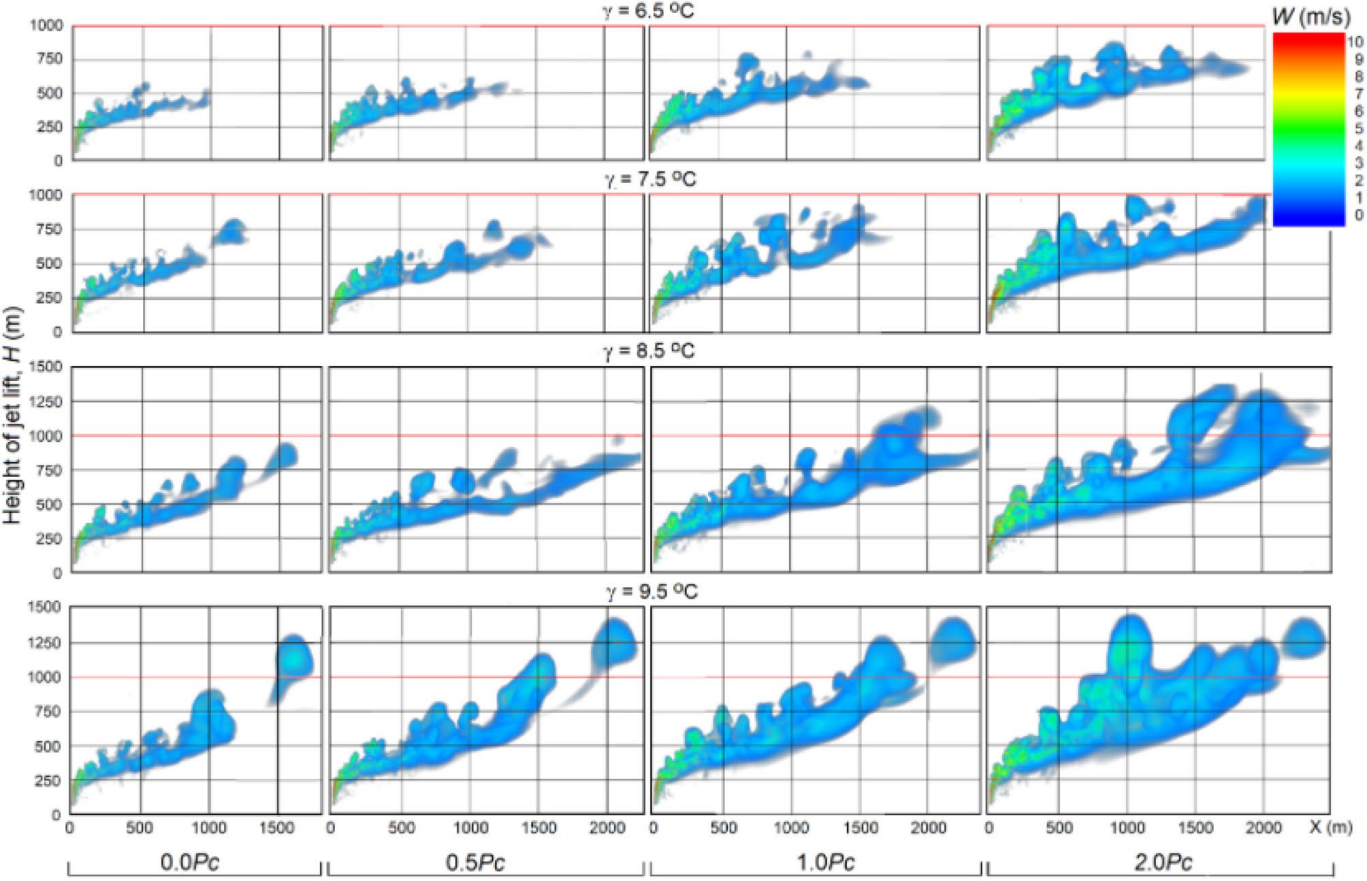
Vertical cross section of artificial updrafts velocity fields *W* (m/s) at energy recharge values *P*_*C*_ = 151 MW, γ = 6.5, 6.5, 8.5 and 9.5 °C/km, vertical wind profile *U* = 1 + 0.005*H* m/s, and *f* = 70%.

**Figure 19:**
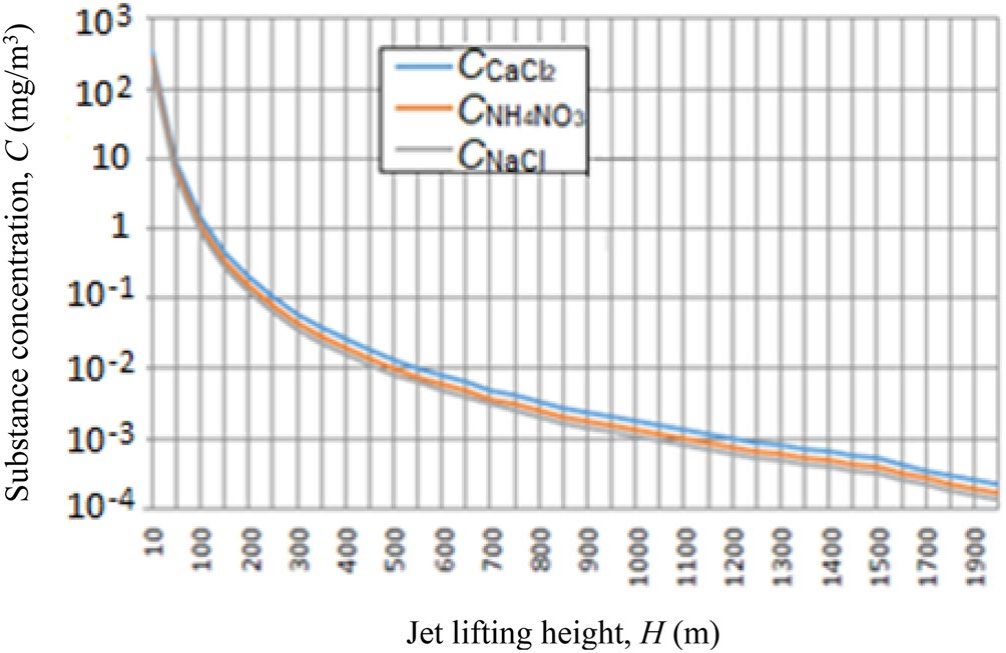
Dependence of hygroscopic aerosol concentration *C* from altitude.

**Figure 20:**
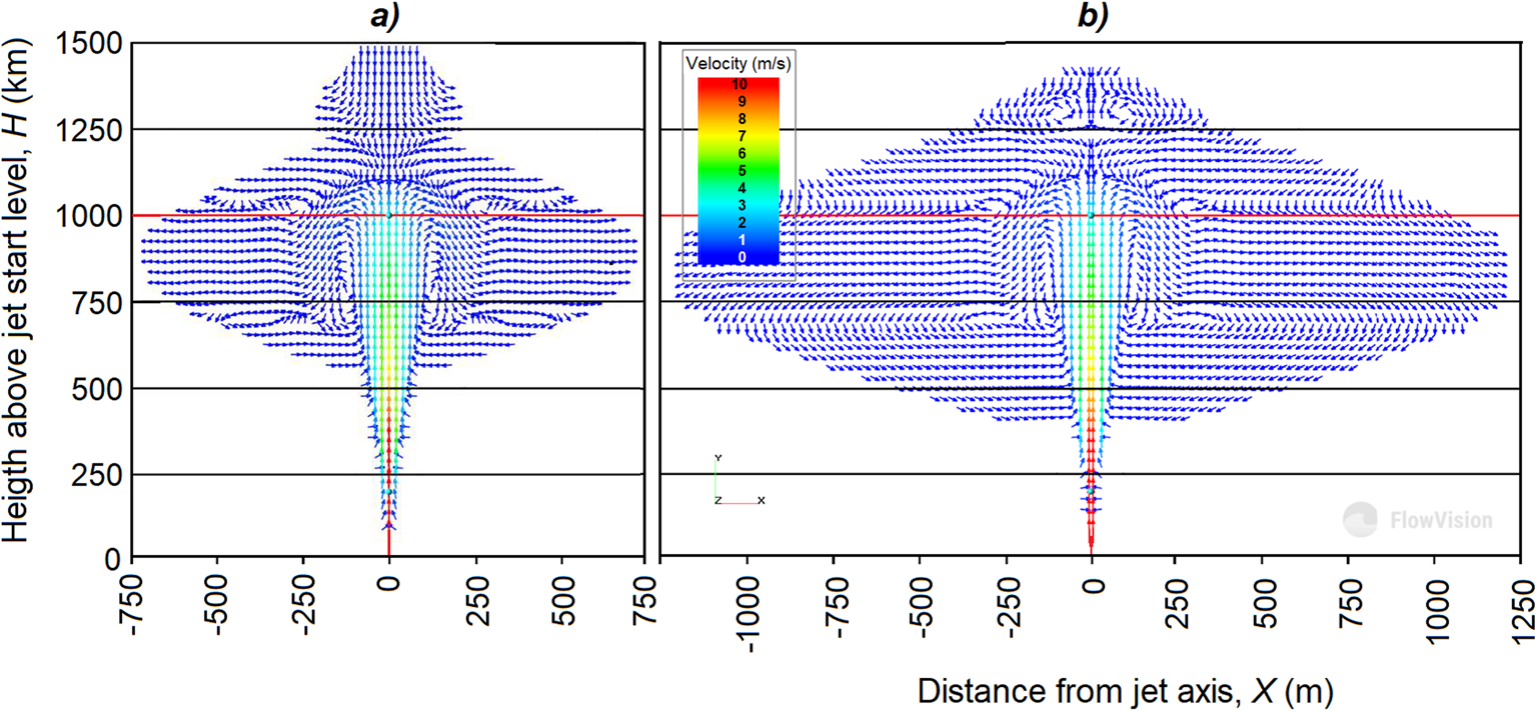
Vertical section of air-aerosol motion in the jet and its surroundings in a windless atmosphere according to the 3-D hydrodynamic model FlowVision: (**a**) without feed; (**b**) with feed.

**Figure 21:**
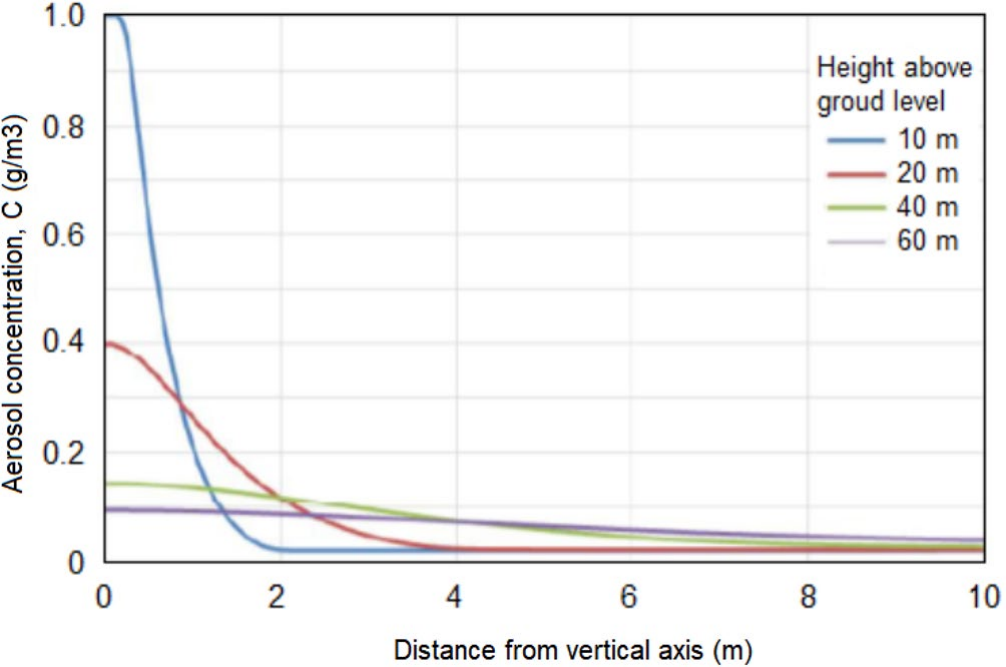
Dependence of aerosol concentration on distance from the jet vertical axis at heights 10 m, 20 m, 40 m and 60 m.

Figures 7, 8, 9, 10, 11, 12, 13, 14, 15, 16, 17, 18, 19, 20, 21 presents the results of calculations of the jet parameters under different atmospheric conditions for the time moment when the quasi-stationary regime of the jet flow was established and the solution convergence was achieved. The moment of convergence was considered to be the moment when the vertical profiles of temperature and velocity of the jet in the layer H < 1 km became almost unchanged. In a windless atmosphere, for the data shown in Figs. 7, 8, such a moment occurred after 20–35 s after the start of the jet action; for the data shown in Figs. 13, 14, 15, 16, 17, 18, 19, 20, 21, after 300–400 s. In the case of wind speed constant in altitude, equal to 1 m/s—at the moments of time shown in Fig. 10. In the case where the wind speed increased with height, (Figs. 11, 12, 17, 18) the time of convergence of the solution to a quasi-stationary flow was about 400–500 s, depending on the variant.

### Compressible set results in the case of a windless atmosphere

The jet at the outlet of the turbojet device of its turning into the zenith has a high (near-sonic) velocity. Therefore, the jet parameters at the stage of flowing into the atmosphere were calculated in the compressible formulation. These calculations showed that due to high turbulence, the jet splits into separate vortices. As a consequence, the instantaneous patterns of radial distributions of updraft velocity *W* (m/s), the jet temperature *T* (°C), and the aerosol mass concentration *C* (g/m^3^) are asymmetrical even in a windless atmosphere (see Fig. 7). The jet disintegrates into individual vortices, so the fields *W*, *T*, and *C* are highly inhomogeneous.

As can be seen in Fig. 8, the jet is torn into separate vortices, which even in a windless atmosphere do not have axial symmetry. Therefore, the two-dimensional mapping of the instantaneous picture of the *W*, *T,* and *C* fields of the jet shows that the jet elements appear to be more elongated in some direction (see Fig. 9). At another point in time, they will be more elongated in another direction. Each of these directions is equally probable, so for quite a long time (about 60 s) the elements of the jet can occupy almost all possible orientations. Symmetry can appear only at summation of turbulent gusts in time on the order of 60 s.

It should be noted that the spraying of water and aqueous solutions of hygroscopic substances leads to a more rapid expansion of the jet and to a certain decrease in speed and temperature. This can be seen by comparing the shapes of the jet in the upper and lower rows in Fig. 8. Thus, the width of the jet in which saline solutions are sprayed (see lower row) is about 2 times wider than the width of the jet without spraying such solutions (see upper row).

### Large-scale atmospheric currents in windy and windless conditions

The input data for calculating the parameters of atmospheric currents at a distance from the aircraft engine are the output data for calculating the compressible jet near the engine installation in the form of averaged radial profiles of the velocity *W*, the temperature increase Δ*T*, and the aerosol mass concentration *C*.

The calculation of the flow in the atmosphere, carried out after such a gluing of the solutions of the problem, shows that the jet acquires a more or less symmetrical shape at altitudes of 500 m and more, after a significant increase in radius and velocity loss.

#### Wind influence

As one might expect, even a weak wind tilts of the jet, and blows it to the leeward side as depicted in Figs. 10, 11. The inclination and drift of the jet are relatively small in the surface layer, where the vertical speed of the jet, *W,* is much higher than the wind speed *U*, and increases with height as the jet speed and the ratio of the jet speed to the wind speed *W*/*U* decreases.

Figure 10 shows the vertical sections of the jet at a wind speed of *U* = 1 m/s at different times from the jet engine start. It can be seen here that above 60 m, most of the jet is carried away from the outflow axis to the leeward side, but 300 s later it rises to 1550 m—almost the same as in the absence of wind (1600 m).

However, situations with such a weak and constant speed wind (*dU*/*dh* = 0) in the entire sub-cloud layer are rarely observed. More often, there are situations when the wind speed increases with height (i.e. *dU/dh* > 0). Figure 11 shows the changes of *W* and Δ*T* fields when wind speed varies by rule *U* = 1 + 0.004*H*. This implies that the wind strongly bends the jet to the leeward side and noticeably reduces the height of its rise. In such situation 70 s after the start of the experiment the jet reaches a height of only 600 m above ground level with an average ascent speed of about 8.6 m/s.

Figure 9 shows the shape of the horizontal section of the fields of instantaneous values of *W*, *T* and *C* at heights of 20, 40 and 60 m in two moment of time: at 25 and 28 s of simulation. At each moment of time, these sections are different due to the influence of high turbulence and are asymmetric relative to the axis even in a windless atmosphere.

The rate of ascent of the jet top in a windless and shallow wind atmosphere is noticeably higher. For example, in a weak wind atmosphere (Fig. 10), in the first 50 s after the start, the jet reaches a height of *H* = 660 m with an average speed of *W* = 13.2 m/s, in the next 50 s, the jet reaches *H* = 1000 m, overcoming 340 m path with an average speed of *W* = 6.8 m/s, and in the next 50 s the jet reaches *H* = 1240 m with *W* ≈ 4.8 m/s, which rises even higher with *W* ≈ 2.8 m/s and less.

As shown in Fig. 11, the jet reaches an altitude of 600 m in 70 s with an average speed of *W* ≈ 8.5 m/s with the presence of wind shear. After 140 s, the jet reaches *H* = 830 m, breaking through a layer with a length of 230 m at a speed of 3.3 m/s, and after 210 s, the jet reaches a maximum height of 860 m, breaking the last 30 m at a speed of 0.43 m/s; i.e., the jet rises with a gradual speed loss. Further action of the source after 210 s does not lead to noticeable changes in the jet shape and height.

With other atmospheric conditions being equal, an increase in the vertical wind gradient *dU*/*dh* from 0 to 0.004*H* m/s leads to a decrease in the jet rise height by almost 2 times (from 1550 to 860 m, although the perturbations of the velocity fields reaches a height of 1000 m); i.e., the presence of even shallow wind (*U* = 1 + 0.004*H*) significantly reduces the jet rise height, which greatly limits the possibility of creating artificial clouds.

In addition to the tilt and lift loss, the wind also deforms the jet in the horizontal section. From Fig. 12, it can be seen that the horizontal sections of the jet at different heights have the shape of an arc enveloping the region of the strongest ascending currents rising above. Thus, the region of maximum flows is flown around by the horizontal wind and lags behind the flow.

From Figs. 11 and 12, it follows that a deformation and a serious decrease in the jet height under the influence of the wind greatly reduces the likelihood of reaching the condensation level and creating artificial clouds. To increase this probability, it is proposed to feed the jet energy with the heat of water vapor condensation on a hygroscopic aerosol, which is massively introduced into the jet at the engine output.

#### Effect of heat condensation jet replenishment

The jet replenishment by increasing the heat of water vapor condensation on a hygroscopic aerosol introduced into the jet at its outlet to the atmosphere leads to an increase in the buoyancy and velocity of the jet ascent, and an increase in the height of its rise and resistance to the wind action. Figure 13 shows, in a windless atmosphere at γ = 6.5 °C/km, that the jet lift height with energy of vapor condensation feed *P*_*C*_ = 5.14 MW is about 10% higher than that of the jet without feed. In this case, the jet temperature without feeding is equalized with the ambient temperature at an altitude of *H*_Δ*T*_ = 775 m, where the jet speed is still 4 m/s, so the jet continues to rise by inertia to an altitude of 1050 m. The jet with feeding rises noticeably higher. Its temperature is equalized with the environment at an altitude of *H* = 860 m, and by inertia it rises to *H* = 1120 m.

Figure 13 shows that the jet temperature and speed decrease as it rises, but the temperature drops faster, and may even be lower than the ambient air temperature up to 1 °C at an altitude where the updraft velocity becomes *W* = 0. That is, in the upper part of the trajectory, the jet continues to rise, having negative buoyancy.

The energetic jet replenishment with the heat of water vapor condensation on a hygroscopic aerosol introduced into the jet at the engine output, as shown in Fig. 14, leads to an increase in the jet lift. This increase at γ = 6.5 °C/km is about 10%, and increases with γ increasing. It also increases with the increase of the power *P*_*C*_ of the feeding source.

Figure 15 shows that, in a windless atmosphere at γ = 6.5 °C/km, doubling the energy feeding power by 2 and 4 times leads to an increase in the jet lift by 6% and 14%, respectively.

When a synthesized core/shell NaCl/TiO2 aerosol is used instead of pure NaCl (Haoran Liang, Linda Zou et al.^26^, the jet feeding increases almost 30 times and reaches *P*_*C*_ = 151 MW. This will lead to significant increases in jet temperature, velocity and height lift and artificial cloud creation potential (see Figs. 16, 17 and 18).

### Influence of temperature lapse rate

Figure 16 shows the results of jet simulation with feeding at different values of temperature lapse rate γ in a windless atmosphere, and Figs. 17 and 18 shows the same with a vertical wind profile *U* = 1 + 0.005*H*. As one would expect, an increase in the γ from 6.5 to 9.5 °C/km leads to a significant increase in the jet height both in a windless atmosphere (see Fig. 16) and under windy conditions (see Figs. 17, 18). Thus, an inclusion of the jet energy recharge leads to an additional increase in the jet lift, which increases rapidly as the feeding energy value increases from 0.0*P*_*C*_ to 2*P*_*C*_ = 302 MW/s.

In windless atmosphere with a humidity over 66%, the jet with energy feeding *P*_*C*_ = 151 MW even at γ = 6.5 °C/km can rise to the height of 1500 m and more (see Fig. 16), and at γ = 7.5, 8.5 and 9.5 °C/km it can reach the level of natural condensation and start the mechanism of cloud and precipitation formation. The presence of wind in the atmosphere sharply reduces the height of artificial updrafts; i.e., wind with a velocity increasing with height (e.g. *U* = 1 + 0.005*H* m/s) leads to the fact that the jet at γ = 6.5 °C/km reaches a height of 800 m only at *P*_*C*_ = 302 MW.

As increases of value γ, the probability of reaching the condensation level increases. For example, it follows from Fig. 17 that at γ = 9.5 °C/km, a jet with an energy supply of *P*_*C*_ = 151 MW can reach a height of 1400 m, while at 70% humidity the height of the natural condensation level will be equal to 1160 m.

Thus, according to the above numerical simulation results, the following favorable conditions are required for a vertically directed jet to reach the condensation level and contribute to the development of convective clouds:

i. Low surface wind speeds and wind shear to condensation levels.
ii. Vertical temperature gradient γ ≥ 8 °C/km.
iii. Increased atmospheric humidity.
iv. Energy feeding of the jet during ascent.

### Environmental safety assessment

The degree of danger of introducing various substances into the atmosphere depends on the method of their introduction, flow rate and hazard class. Table 3 shows data from the passports of substances used in the implementation of the method under consideration; i.e., the number in the Chemical Abstracts Service (CAS), the number in the register of the European Agency (EU), the hazard class, maximum permissible concentrations (MPC) and the consumption of substances per experiment and per 1 s, as well as the initial concentration in the jet stream. The MPC of the used substances in the soil is 150–200 mg/kg, in the water of open reservoirs around 45 mg/l, while in the atmospheric air it is 0.2 mg/m^3^.

These substances are introduced into the vertically directed jet by spraying their aqueous solutions close to saturation. In a 30 min experiment, it is planned to spray about 1800 kg aqueous solution of each substance. The consumption of these substances depends on their solubility and the density of aqueous solutions. Table 3 shows the total amount of substances sprayed in one experiment and the flow rate per second. Since the volume of the reactive gas flow is around 200 m^3^/s, the initial concentration of substances introduced into the reactive gas stream will be about 1000 mg/m^3^ (see the last column of Table 3).

As it rises, the jet expands and spreads, the diameter of the jet reaches 1.0 km, and its height is 1.2 km. Our calculations show (see Fig. 19) that already at an altitude of 1 km, the concentration of the introduced substances is about 10^–3^ mg/m^3^.

Numerical experiments using the 3-D hydrodynamic model of FlowVision also shows that the artificial aerosol concentration rapidly decreases as the jet rises due to the jet expansion and mixing with the ambient air, and at an altitude of 50–100 m it decreases tenfold. The jet volume can reach *V*_*J*_ = 10^9^ m^3^ even in a windless atmosphere (see Fig. 20). In these conditions, in the case of uniform turbulent mixing, the average concentration of aerosol introduced into the jet will be *N*_*t*_ = *M’*/*V*_*J*_ = 2⋅10^–4^ mg/m^3^. The wind action will further reduce its concentration. In addition, aerosol concentration decreases from the jet center to its periphery as shown in Fig. 21.

Thus, the introduction of hygroscopic substances into a jet stream leads to their removal to great heights, dispersal in large spatial volumes and deposition over vast spaces. As a result, the concentration of the introduced substances in the air, water of open reservoirs and soil will be lower than the MPC, and the norms for the consumption of fertilizers in agriculture (from 3 to 40 g/m^2^).

## Discussion

The possibility of creating artificial clouds and precipitation by local heating of the surface atmosphere using buoyancy jets depends mainly on the jet temperature, speed and radius, which are determined by the engine power; and also on the parameters of the atmosphere, including their values near the ground and the vertical profiles of temperature, air humidity and wind speed.

**Table 3:** Hazard class and consumption of hygroscopic substances.

Substance name	No Gas	No ES	Hazard class by		MPC, mg/m^3^	Expense for 1 experiment, *M* (kg)	Expense per 1 s, *M’* (g/s)	Initial volume concentration, *N*_*V*_ (mg/m^3^)
			UN list	Russian standard				
Calcium chloride	7774-34-7	233-140-8	Not any	III	0.2	530	295	1472
Nitroammophosk				Not any	0.2	400	222	1110
Foot salt	7647-14-5	231-598-3		Not any	2.069	360	200	1000
Carbamide A	57-13-6	200-315-5		IV	0.2	340	189	940

Clearly, increasing the power of the jet engine makes it easier to solve the problem, but this is limited by the technical characteristics of jet engines from decommissioned aircraft. In addition, an increase in engine power cannot lead to the same proportional increase in the effect as fuel consumption.

Another way to achieve this goal is to select favorable atmospheric conditions when the engine power may be sufficient for the jet to reach the level of natural condensation and trigger the cloud formation mechanism.

In this work, in contrast to earlier attempts to create artificial clouds using meteotrons (Dessens 1961)^15–20,25^, it is proposed to increase the target potential by introducing energy supply to the vertically directed buoyancy jet by the heat of water vapor condensation on a hygroscopic aerosol, which is introduced near the jet engine.

To assess the efficiency of this approach, a 3-D numerical simulation of the vertical jet motion in the atmosphere with and without energy recharge, depending on the atmospheric characteristics, was carried out on the basis of the CFD FlowVision software package suite. The numerical simulation results showed the following:

1. The motion of a high-speed jet in a real atmosphere has a complex turbulent character due to its high speed. The fields of jet vertical velocity and excess temperature are extremely inhomogeneous and, even in a windless atmosphere, pulsate around their axis.
2. As it rises, the jet expands, due to the entrainment of ambient air, and loses superheat and the rate of ascent. In this case, the jet temperature decreases faster than the speed. As a result, in the upper part, the jet continues to rise by inertia even when its temperature equals the ambient air temperature, and then becomes even lower by 1 °C. Spraying water and aqueous solutions of hygroscopic substances leads to a more rapid expansion of the jet, a decrease in its speed and temperature.
3. The wind in the surface layer of the atmosphere leads to the jet deformation in the vertical and horizontal plane tilts and blows the updraft top to the leeward side for hundreds of meters. In the horizontal section, the jet takes the form of an arc covering the region of maximum updrafts, which lags behind the ambient air flow by analogy with the lagging behind the leading flow of the updrafts region and the powerful radar echo of convective storms^29^. The presence of even shallow wind (*U* = 1 + 0.005*H*) almost halves the jet height, which greatly limits the possibility of creating artificial clouds. This is one of the reasons for the modest results previously obtained^18,21^ in experiments with meteotrons.
4. High values of temperature lapse rate γ favors increase in the jet lifting height. Calm atmosphere with γ from 6.5 to 9.5 °C/km increases the jet lift by 25%. In the presence of wind, γ increases from 6.5 to 9.5 °C/km, despite significant deformation and drift of most of the jet downwind (by 500–1000 m), the jet rise increases by 60% (500–700 m).
5. Numerical experiments also showed that the inclusion of the jet feed with the heat of condensation *P*_*C*_ leads to a significant increase in the jet lift and its resistance to the destructive effect of the wind. Increasing the energy feeding by 2 and 4 times, increases the jet lift by 6% and 14%, respectively.
6. It is more effective when spraying a novel two-layer core/shell NaCl/TiO_2_ hygroscopic aerosol into the jet, which is capable to absorb water vapor 295 times more of its dry mass and increasing the amount of *P*_*C*_ feeding by 30 times compared to the similar aerosol of pure NaCl. Such an increase in the energy feeding at air humidity over 66% can increase the jet lift so much that the creation of artificial clouds will become quite real.
7. On the basis of numerical experiments, it was also established that an increase in the jet engine installation height above sea level, for example, by 1000 m leads to an additional increase in the jet rise height, which is apparently explained by a decrease in the resistance of the medium and an increase in the temperature difference between the jet and the medium, where the higher the colder.
8. An increase in air humidity also leads to an increase in the jet lifting height even without condensation of water vapor due to the fact that moist air is lighter than dry air.
9. In general, the results of modeling the motion of a jet stream with energy replenishment in the atmosphere are encouraging in the possibility of creating artificial updrafts that reach the level of condensation. It can, under certain atmospheric conditions, lead to the formation of convective clouds with precipitation.

In the case of feeding the jet with condensation heat on NaCl/TiO_2_ aerosols, favorable conditions for creating artificial convective clouds can be.

i. Surface wind speed *U* ≤ 2 m/s and wind shear *dU/dh* ≤ 0.005* h*;
ii. Vertical gradient of air temperature γ ≥ 7.5 °C/km;
iii. Air humidity *f* > 66%;
iv. Absence of powerful temperature inversion layers.

With weaker recharge, the range of favorable atmospheric conditions is narrowed and the frequency of occurrence of such conditions in arid regions is significantly reduced.

## Conclusions

To study the possibility of creating artificial clouds and precipitation using a vertically directed buoyancy jet saturated with a hygroscopic aerosol, adaptation and testing of the CFD FlowVision software package suite was carried out and 3-D simulations of the jet propagation in a free atmosphere with and without energy replenishment using latent heat of water vapor condensation were carried using Navier–Stokes system of motion equations.

Numerous numerical experiments have been carried out to study the structure of the velocity and temperature fields, as well as the concentration of aerosol introduced into the jet when it enters the atmosphere. It was shown that the energetic replenishment of the buoyancy jet leads to an increase in the ascent height in the atmosphere, which increases the potential for penetrating of the inversion layers, reaching the level of natural condensation, and triggering the cloud formation mechanism. Strong winds, wind shear and large inversion layers are severe obstacles to stimulating artificial convection.

The creation of artificial clouds and precipitation with the help of a vertically directed reactive jet with replenishment is possible under favorable atmospheric conditions, which are close, but have not yet reached the conditions of natural development. The range of favorable conditions for stimulating cloud convection is significantly expanded due to the use of a hygroscopic aerosol such as core/shell NaCl/TiO_2_.

In conclusion, it should be noted that the presented results are preliminary, since in the numerical experiments linear vertical profiles of wind speed, air temperature and humidity were set, without inversion layers.

## Data Availability

All data generated or analyzed during this study are included in this published article.
